# Generic and queryable data integration schema for transcriptomics and epigenomics studies

**DOI:** 10.1016/j.csbj.2024.11.022

**Published:** 2024-11-19

**Authors:** Yael Tirlet, Matéo Boudet, Emmanuelle Becker, Fabrice Legeai, Olivier Dameron

**Affiliations:** a Univ Rennes, Inria, CNRS, IRISA, 35000, Rennes, France; b IGEPP, INRAE, Institut Agro, Univ Rennes, 35653, Le Rheu, France

**Keywords:** Multi-omics analysis, Data integration, Integration schema, Semantic web

## Abstract

The expansion of multi-omics datasets raises significant challenges for data integration and querying. To overcome these challenges, we developed a generic RDF-based integration schema that connects various types of differential -omics data, epigenomics, and regulatory information. This schema employs the FALDO ontology to enable querying based on genomic locations. It is designed to be fully or partially populated, providing both flexibility and extensibility while supporting complex queries. We validated the schema by reproducing two recently published studies, one in biomedicine and the other in environmental science, proving its genericity and its ability to integrate data efficiently. This schema serves as an effective tool for managing and querying a wide range of multi-omics datasets.

## Highlights

• Creation of a generic, queryable and extensible RDF data integration schema.
• Validation through data integration and replication of results from recent biomedical and environmental studies.
• Along with the integration schema, we provide a SPARQL query library for analysis.

## Graphical abstract

**Figure:**
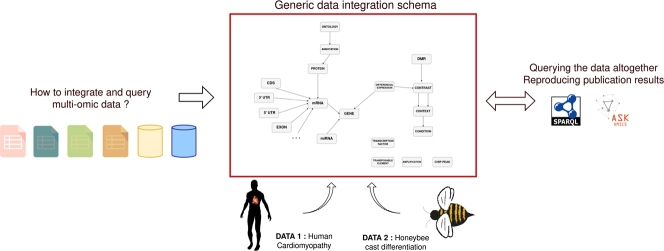

## 1 Introduction

**Fig. 1:**
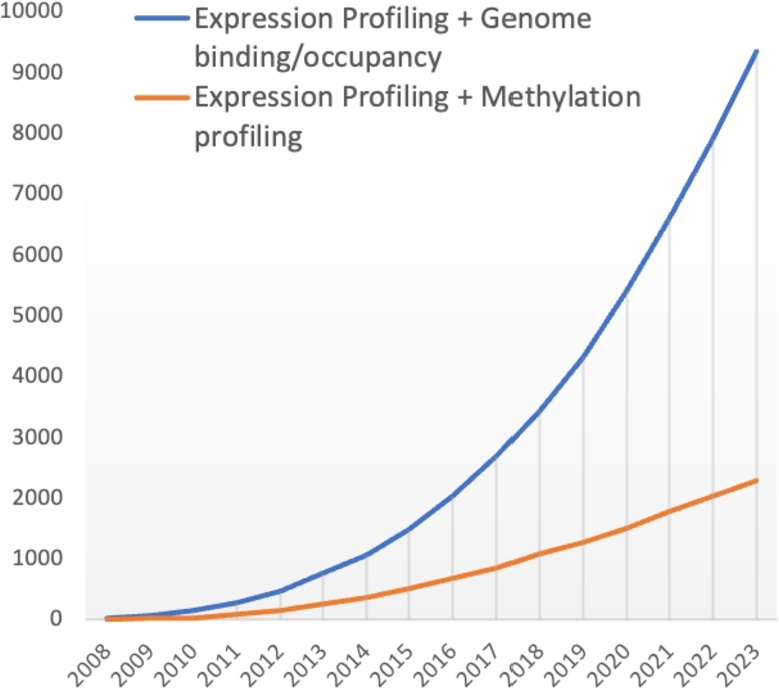
Number of GEO Datasets with 2 distinct -omics modalities (multi-omics datasets) available each year between 2008 and 2023.

Multi-omics data are widely used to identify key factors in human health [1], [2], [3], [4], [5], ecological and environmental studies [6], [7], or evolution [8], [9], [10], [11]. Particularly, the number of studies coupling transcriptomics and epigenomics has significantly increased over the last 15 years, as it can be measured in the Gene Expression Omnibus (GEO) datasets database (Fig. 1).

Transcriptomics studies how messenger and non-coding RNAs are expressed in an organism under specific conditions. It is usually quantified using RNA sequencing (RNA-Seq) and estimated with normalized read counts in a library. This approach identifies candidate genes that show a significant change in expression level between contrasted conditions.

Epigenomics studies the mechanisms that regulate gene expression under specific conditions. Epigenomics includes regulation by short non-coding RNA such as miRNA, siRNA, or piRNA [12], [13], whose expression dynamic can be studied with dedicated sRNA-seq protocols [14], [15], [16]. Common epigenomics analyses also involve DNA methylation, histone modifications, and chromatin accessibility. Several sequencing methods, such as bisulfite sequencing [17], [18], ChIP-Seq [19], [20], [21], FAIRE-Seq, and ATAC-Seq [22], [23], can precisely locate these epigenetic modifications.

Simultaneously studying gene expression with genome binding or methylation offers critical insights into the mechanisms of gene regulation, linking epigenetic changes to gene activity and providing a dynamic, integrated view of cellular function. This approach helps identify biomarkers and therapeutic targets, enhancing our understanding of complex diseases.

Depending on the project context, several strategies for multi-omics dataset integration can be applied, and recent publications developed classifications for these integration strategies [24], [25], [26]. The proposed classification mainly distinguishes three types of integration based on the order of the fusion and analysis phases. The *early integration* methods combine all input matrices into a single dataset before analysis. This approach increases the number of variables in the dataset while not accounting for the differences in the different -omics distributions. Second, *intermediate integration* where the input datasets are jointly represented into common and omic-specific representations before analysis (e.g., matrix factorization). Finally, in the *late integration* methods, the datasets are analysed separately for each -omics, and the results are then integrated. This strategy is not optimal to capture inter-omics interactions, but allows to re-use previous analysis. Late integration methods are also ideally suited for research consortia focusing on a similar biological subject and comprised of experts with their respective areas of specialization.

In this article, we focus on late integration methods for integrating results from transcriptomics studies (lists of differentially expressed genes -DEGs- between conditions), results from epigenomics studies (genomic regions involved in gene regulation, measures of small RNA expression under similar conditions), and genomic features that precisely locate genes on a reference sequence. These datasets are distinct pieces of a larger puzzle that must be integrated in order to propose new hypotheses or validate existing ones. Whenever possible, these data should be supplemented by knowledge such as functional annotation of genes/proteins, literature, or previous results such as preliminary research or comparisons with closely related organisms.

**Fig. 2:**
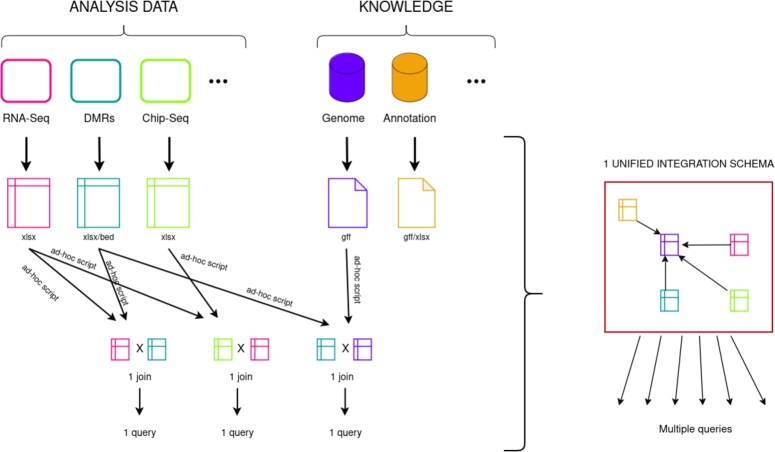
**Our vision of data integration challenge.** Multi-omic datasets combine the results of different single -omics, including differentially expressed genes, differential methylation regions (DMRs), histone modification sites... as well as additional knowledge such as genomic features and annotations. Various files (xlsx, GFF, bed...) need to be joined two by two using *ad-hoc* scripts to answer one question. It leads in a large number of *ad-hoc* scripts, often too specific to be reused. We propose a unique generic integration schema that links all those data and knowledge, and can then be queried to answer several questions.

Late integration often involves computer scripts to parse and connect the output files of each single-omics analyses, as illustrated in the left panel of Fig. 2. For example, scripts can be written in Python with Pandas or in R with Tidyverse, or using specific tools such as bedtools [27]. This can be interpreted as a limitation for a variety of reasons. To begin, a basic understanding of programming is necessary. Second, while the raw data from the analyses is usually available on dedicated platforms such as GEO or Zenodo, these critical scripts for the conclusion of the articles are not necessarily shared, thus limiting the Accessibility and Interoperability of the FAIR principles (Findability, Accessibility, Interoperability, and Reproducibility) [28]. When available, these *ad-hoc* scripts are often highly dependent on the precise biological question they were developed for, thus preventing their re-use for other studies with a similar experimental plan. In the end, this multiplicity of *ad-hoc* scripts shared (or not) independently for each research article and rarely re-used increases the potential number of hidden bugs, *i.e.* bugs undetected by the scientific community over time.

Early initiatives, such as InterMine [29] or BioMart [30], presented dedicated tools to integration difficulties but failed to scale up to the multiplicity of resources and the diversity of demands. There is thus a need to develop more generic and standardized methodologies for integrating multiple single-omics datasets. Initiatives based on graph-oriented databases have been introduced in recent years, such as Ortho_KB [31] and Pantools [32]. These systems provide genericity and standardization, but are not optimal for handling ontology-based annotations or connecting external information sources.

The semantic web provides a generic framework for integrating heterogeneous and complementary data, which has been widely implemented in life sciences [33], [34], [35], with the potential to integrate and reason on ontologies. The Semantic Web technical stack combines Ressource Description Framework (RDF) for describing data, RDF Schema (RDFS) for defining the structure of these descriptions, Web Ontology Language (OWL) for defining explicitly the knowledge underlying the descriptions, and SPARQL Protocol And RDF Query Language (SPARQL) for querying the previous three formalisms [36].

In this study, we provide a new RDF-based data schema that can be used to integrate and query a large number of studies combining transcriptomic dataset, epigenetic datasets, and annotations. We illustrate the schema by reproducing two recently published studies, one in biomedicine focused on cardiopathic hypertrophy in humans, and the other in environmental science, focused on the epigenomics of castes in bees. With these use-case, we prove the genericity of the proposed schema, and its ability to integrate data efficiently to reproduce published results.

## 2 Material and methods

In this section, we first introduce the Semantic Web principles and the RDF schema proposed. We then briefly present the Askomics software, which has a convenient graphical interface, that we used to design the data schema, populate the data schema, and query the data. The Askomics instances generated for each use-case are available at: https://hcm.askomics.org/ and https://honeybee.askomics.org/, populated with the data, and showing all the queries used to reproduce the use-case publications.

In this section we also present the two datasets we used as use-cases. These are extracted from two recent papers that were written by independent research teams (not related to ours). We choose two examples with very different topics (health vs. environmental application) as well as different omics data to highlight the genericity of our schema (differential expression, differential methylation and transcription factors regulation for health dataset, *vs.* differential expression, transcription factor binding sites, chromatin accessibility and conformation for the environmental dataset).

### 2.1 Semantic web and askomics

#### 2.1.1 Semantic web principles

In the Semantic Web framework, each entity (e.g. gene, transcript, experimental condition) is identified by its Uniform Resource Identifier (URI). To achieve integration, each dataset that refers to the same entity utilizes its URI. The Resource Description Framework (RDF) representation of each dataset is a set of triples that describe the fundamental properties of each entity, relationships to other entities, and the classes to which they belong (see RDFS below). An RDF dataset is therefore a labeled directed graph. If two datasets share some URIs, their graphs can seamlessly be merged.

An RDF schema (RDFS) is a special dataset, also represented in RDF, that specifies (1) the URIs for entity classes (e.g., gene, transcript, miRNA, lncRNA, etc.), (2) the relations between some of these classes (e.g., the miRNA and lncRNA classes are subclasses of the transcript class), (3) the URIs for RDF triple relations, and (4) domain and range constraints for these relations. RDFS therefore specifies the structure of RDF datasets. RDF datasets and schemas are denoted by RDF(S).

SPARQL is the query language used for RDF(S) datasets.

#### 2.1.2 RDF schema for integrating transcriptomics and epigenomics studies

**Fig. 3:**
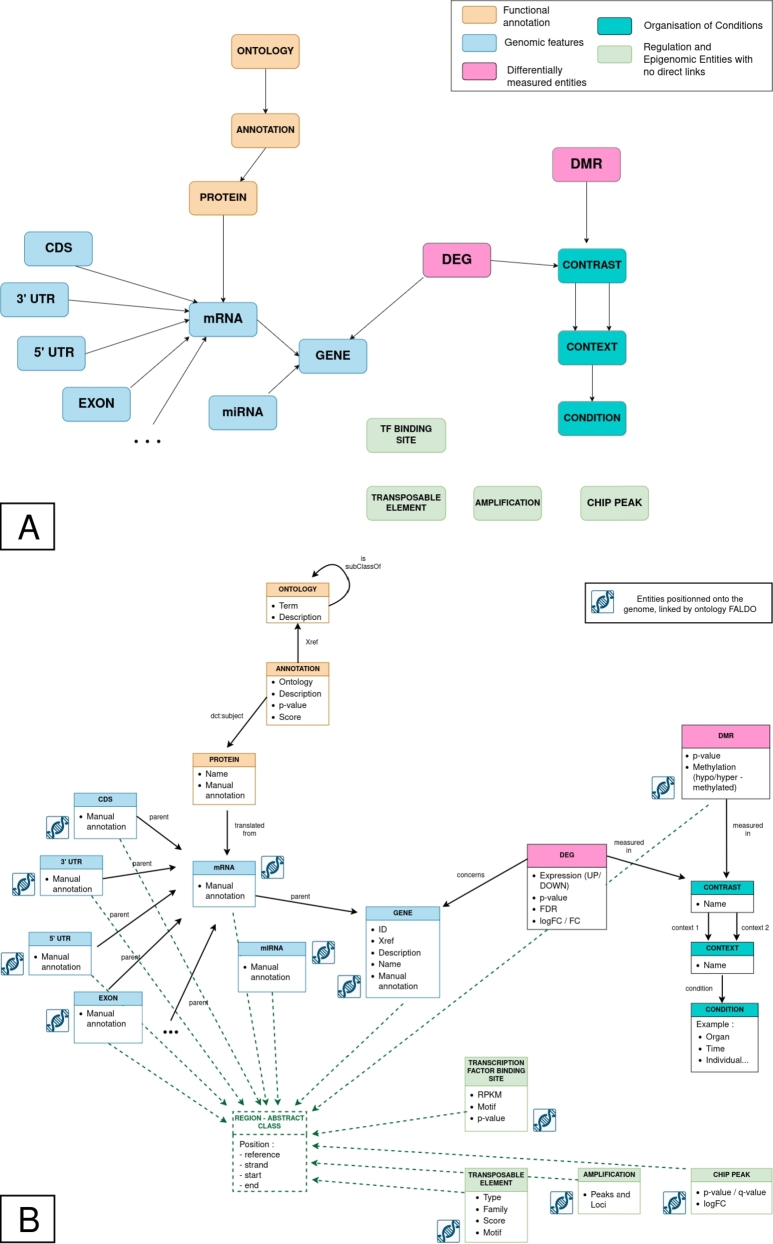
**Generic integration schema. (A)** Simplified view of the integration schema, including entities corresponding to genomic features (light blue boxes), entities corresponding to functional annotations (orange boxes), entities describing the experimental plan (turquoise boxes), entities describing differentially expressed entities (pink boxes), and other entities related to regulation that can be located on the genome (e.g. transposable elements, transcription factors binding sites, amplified regions... in light green boxes). DEG: Differentially Expressed Gene, DMR: Differential Methylation Region. **(B)** Detailed view of the integration schema. For each entity, some attributes are listed. Entities positioned on the genome, identified by a chromosome symbol, are linked to the “Region” abstract class (FALDO relations).

To accurately represent genomic features, we included all entities usually found in GFF format^1^: gene, transcript/mRNA, exon, CDS (light blue boxes in Fig. 3)... In our data schema, we introduce parent-type relations between these items. For example, a gene entity is a parent of another mRNA entity, which in turn can be a parent of an Exon, CDS, 5'UTR, or 3'UTR. According to the GFF, all of these elements are precisely located on the genome, therefore all these entities share common attributes: chromosome, strand, start, and end. The Feature Annotation Location Description Ontology (FALDO) [37] allows entities with these properties to be automatically connected with FALDO relations, and thus easily queryable.

Our data schema enables the incorporation of knowledge about protein functional annotation, such as Gene Ontology (GO) terms^2^ or Kyoto Encyclopedia of Genes and Genomes (KEGG) identifiers.^3^ This knowledge is structured in three entities: ontology, annotation, and protein, which we linked directly to mRNA (orange boxes in Fig. 3). Because our schema is based on semantic web principles, the sub-class relation between the different terms of the ontologies will be automatically accounted for.

The data schema is dedicated to integrate data highlighting differences between conditions, so we must provide a data schema that accurately defines the conditions and contrasts that produce these differences. We defined three entities (turquoise boxes in Fig. 3): the Condition entity with the different experimental conditions, the Context entity that aggregates Conditions and will be compared as described in the Contrast entity.

Transcriptomics studies such as RNA-Seq, miRNA, and some other -omics studies such as measurements of genome methylation, present differential entities when compared in a Contrast (pink boxes in Fig. 3). These -omics entities are either already located on the genome (FALDO [37]) or can be linked to existing entities that are. For others -omics studies and regulation entities such as ChIP peaks, Amplifications, Transcription Factors or Transposable Elements, we added them as entities with only FALDO links (light green boxes in Fig. 3).

#### 2.1.3 Askomics general architecture

The Semantic Web has a long history of technical successes for integrating heterogeneous and complementary life science datasets [35]. However, the learning curve for expressing end-user data in RDF, for combining them with other datasets, and for crafting the SPARQL queries has understandably hampered its adoption by life science experts. To make it easier for a large audience to utilize our integration schema, we investigated how dedicated tools may assist users in integrating their datasets and composing SPARQL queries to exploit them.

AskOmics^4^ is a data integration and querying tool based on Semantic Web technologies. Through a web interface, users are able to integrate both local and remote datasets of various formats, and formulate biologically-relevant questions iteratively via a querying interface automatically tailored to the integrated data.

AskOmics consists of two functional modules, each consisting of a user interface interacting with specific data treatment and storage procedures.

The first module (2.1.4) provides a framework for integrating selected heterogeneous datasets, by populating a local triplestore with both the data itself, and its automatically generated data schema. Optionally, the data schema of remote triplestores can be integrated and linked to the local data schema.

The second module (2.1.5) enables the user to graphically design complex queries by interacting with partial views of the data schema. This automatically generates the corresponding SPARQL code and send the query to the triplestore.

#### 2.1.4 Data integration module

Through this module, users can upload and integrate datasets in a combination of formats including tabular files, biology-specific file types like GFF and BED, and typed RDF data.

A specific integration pipeline is offered for each data format, allowing the user to fine-tune the data conversion and resulting data structure (links between entities, attribute type, remote endpoints). At the end of the pipeline, AskOmics will automatically convert the data and integrate both the data itself and its data schema into the local triplestore.

In our use-cases, the different tables as well as the GFF3 including the annotation of the genomes were integrated in Askomics. The cross-links between these tables were automatically detected based either on the genome positions (FALDO [37] relations), or based on columns names that match the following format: relation@OtherTableEntity (example: differentiallyExpressedIn@Contrast).

#### 2.1.5 Query generation module

By using the integrated data schema, AskOmics is able to provide a tailored querying interface to the user. After selecting a starting entity, users are able to view all the entity attributes and relations. Through the web interface, it is possible to set constraints on both attributes and relations. Users can then ‘jump’ to a related entity to add additional constraints, iteratively and interactively building a complex query to answer their biological questions. Once the query is ready, it can be submitted to the triplestore, or stored and shared with other users to be improved upon.

We generated all queries for use-case 1 and use-case 2 with the dedicated Askomics module. Queries have been saved and are available on the Askomics instances (https://hcm.askomics.org/ and https://honeybee.askomics.org/).

### 2.2 Use-case 1: biomedical data about hypertrophic cardiomyopathy

We chose a study by Gao et al. [38]: *“Integrative analysis of transcriptome, DNA methylome, and chromatin accessibility reveals candidate therapeutic targets in hypertrophic cardiomyopathy”* as our first use case. Because the mechanism underlying cardiac remodeling in hypertrophic cardiomyopathy (HCM) is poorly understood, the authors investigated the subject using a multi-omics approach, which included DNA methylation, chromatin accessibility, and gene expression. We downloaded the results of these analyses from the supplementals and integrated them into our generic, queryable data schema.

The dataset contains data from healthy adults (Control), adult patients (HCM), and healthy fetuses (Fetus). The data types integrated include differentially expressed genes (DEGs) and differentially expressed lncRNAs in HCM *vs.* Control, co-regulated DEGs, differential methylated regions (DMRs) and enriched motifs of transcription factors (TF). The human genome used in this study is GENCODE human genome hg19.

From the supplementary tables, we created tables to describe experimental Conditions, Contexts which are a composition of Conditions, and Contrasts which represents the comparison of two Contexts).

Table S3 of Gao et al. [38] includes four spreadsheets that list separately protein-coding genes and non-coding RNA down- or up-regulated between HCM and Control, with related variables: gene ID, gene name, gene type (protein coding), fold-change, p-value, and corrected p-value (FDR). Before integrating in Askomics, we combined all of these sheets into a single DEG table and added two columns to reflect the corresponding Contrast and regulation sense (UP or DOWN).

In addition, the authors included two lists of co-regulated genes in the supplementary table S5, *i.e.* genes that were dis-regulated in both HCM and Fetus as compared to Control, and had the same regulation sense (UP or DOWN). These sheets were combined with basic information: gene ID, gene name, gene type, regulation sense (UP or DOWN), and then integrated into Askomics.

Epigenomics results presented in the supplementary table S4 of [38] are composed of two tables with the differentially methylated regions (DMRs), one for hyper-methylated regions between HCM and Control, and the other one for hypo-methylated regions between HCM and Control. For each region, the features are the position on the genome (chromosome, start, end) and the associated gene. We concatenated the two tables and added two columns to show if the region was hyper- or hypo-methylated and to precise the Contrast resulting in differential methylation.

For a subset of genes corresponding to nucleosome-depleted regions (NDRs), the regions were screened for transcription factor binding sites. This information is split in six tables in the supplementary file S6 of [38]. We also concatenated these tables, and added new columns to indicate the related Condition, and distinguish proximal and distal regulations.

Scripts for concatenating tables are accessible on the git repository: https://github.com/ytirlet/Generic-and-Queryable-Data-Integration-Schema.

### 2.3 Use-case 2: environmental data about honey bee cast differentiation

As a second use-case, we selected a study by Zhang et al. [39]: *“The diverging epigenomic landscapes of honeybee queens and workers revealed by multi-omic sequencing”*. We downloaded the results from the supplementary materials, concatenate tables when information was splitted in several tables, and then integrate them into our schema using Askomics.

The data were collected from queen (Q) or worker (W) bees, at 2 or 4 days. Two Contrasts are studied: Q *vs.* W at 2 days (2Qvs2W) and Q *vs.* W at 4 days (4Qvs4W).

The authors provide a list of differential elements for each Contrast, including differentially expressed genes (DEGs) from RNA-Seq and conformation switch predictions from Hi-C analysis. The data also includes chromatin accessibility information from ATAC and ChIP-Seq peaks. In the original study [39], functional enrichment was explored to identify KEGG IDs over-represented in the DEGs of each Contrast. The authors also included annotations from previously published articles to highlight genes known to be involved in cast differentiation as well as targets of the transcription factor Aft1.

The genome used in this work is Amel_HAv3.1 388 (GCF_003254395.2).

Based on the experimental design of use-case 2, we constructed tables to describe experimental Conditions, Contexts, and Contrasts.

The authors chose to present DEGs splitted into four tables depending on the Contrast (2Qvs2W or 4Qvs4W) and regulation sense (UP or DOWN regulation). As in 2.2, these tables were concatenated and two columns were added to precise the Contrast and the sense of regulation (UP or DOWN) of each entry.

Hi-C analysis results are also presented in four tables based on the Contrast (2Qvs2W or 4Qvs4W) and conformation switch (A → B or B → A). These tables contain regions of 100000 base pairs delimited by their genomic location and a score. These four tables were concatenated into a single one, with two columns added to reflect the Contrast and conformation switch of each entry.

ATAC unique peaks were provided in four tables for the four Conditions: 2Q, 2W, 4Q, and 4W. The data include the position of the peak, fold-change, p-value, q-value, and annotations. We concatenate the four tables and added a column to indicate the corresponding Condition for each entry. The ChIP data were processed the same way.

The two remaining tables, containing a list of genes known to be involved in cast differentiation and a list of Aft1 targets, were directly included.

Scripts for concatenating tables are accessible on the git repository: https://github.com/ytirlet/Generic-and-Queryable-Data-Integration-Schema.

## 3 Results

### 3.1 Global multi-omic generic integration schema

We propose a generic schema to integrate genomic features, transcriptomic and epigenomic data together. The integration framework is organized into five major interconnected blocks that structure the genomic and epigenomic data. These five blocks are dedicated to the different data to be integrated and represented in different colors in Fig. 3: genome features (light blue boxes), protein annotations (orange boxes), experimental design (turquoise boxes), differentially measured entities (pink boxes), and regulation/epigenomic entities (light green boxes). These different blocks are described below. Sections 3.2 and 3.4 will provide additional examples for using this framework.

To precisely describe genomic features, we included all entities usually found in GFF format (gene, mRNA, CDS...). These entities are illustrated with light blue boxes in Fig. 3. Relations of type parent between entities are represented with arrows between light blue boxes, while FALDO relations are not represented with arrows in Fig. 3-A. Instead, entities that can be linked with FALDO relations are indicated with a genome logo and are linked to a Region abstract class (green box and dashed green arrows in Fig. 3 B).

Our data schema allows to include knowledge about functional annotation, such as Gene Ontology terms or KEGG IDs. The corresponding entities are indicated with the orange boxes in Fig. 3. This functional annotation block is connected to the genomic features block by linking proteins to the mRNA they are derived from.

Because our data schema is dedicated to integrate data highlighting differences between experimental conditions, we have to propose a schema that accurately describes the experimental plan. The experimental plan is finely described with three entities illustrated in turquoise boxes in Fig. 3: Condition, that represent one precise experimental condition, Context, that groups several Conditions, and Contrast, that compares several Context. The Context entity was created for a higher flexibility: in -omics studies with a sophisticated experimental plan and more than one parameter varying, Conditions are often grouped differently to explore different Contrast. Examples will be presented in 3.3 and 3.5.

Transcriptomic and other -omics studies often present results as list of differentially measured elements given a Contrast. For example, these include analysis of differentially expressed genes (DEG) or differentially methylated regions (DMRs). These analyses are integrated in our data schema in pink in Fig. 3 A, and linked to the experimental plan block *via* the Contrast they are measured in. Whenever possible, these are also connected to the genomic features block given their location on the genome using FALDO relations [37].

The fifth block of entities, shown in light green in Fig. 3, includes additional epigenomic and regulatory entities, including ChIP peaks, amplifications, transcription factors binding sites, and transposable elements. All these entities are connected to the integration schema *via* FALDO relations. These FALDO relations are hidden in Fig. 3-A, while Fig. 3-B highlights their connection to the rest of the schema *via* the connections to the Region abstract class.

The goal of this schema is to be generic enough to work with any study combining transcriptomics and epigenomics. The Askomics tool was used to generate the RDF schema, and then to populate and query it. Each use-cases will populate distinct areas of the schema.

### 3.2 Populating the data schema for biomedical data about HCM (use-case 1)

We first illustrate how to use our data schema with a use case involving biomedical data. The data are related to a recent paper by Gao et al. [38] entitled “*Integrative analysis of transcriptome, DNA methylome, and chromatin accessibility reveals candidate therapeutic targets in hypertrophic cardiomyopathy.*” The authors of this publication aim to characterize hypertrophic cardiomyopathy (HCM) by investigating both genomic and epigenomic regulation.

The experimental plan of this study involves three cohorts: healthy adults (Control), adult patients (HCM), and healthy fetuses (Fetus). The results presented in [38] include differentially expressed genes (DEGs) and differentially expressed lncRNAs in HCM *vs.* Control, co-regulated DEGs, differential methylated regions (DMRs) and enriched motifs of transcription factors (TF).

**Fig. 4:**
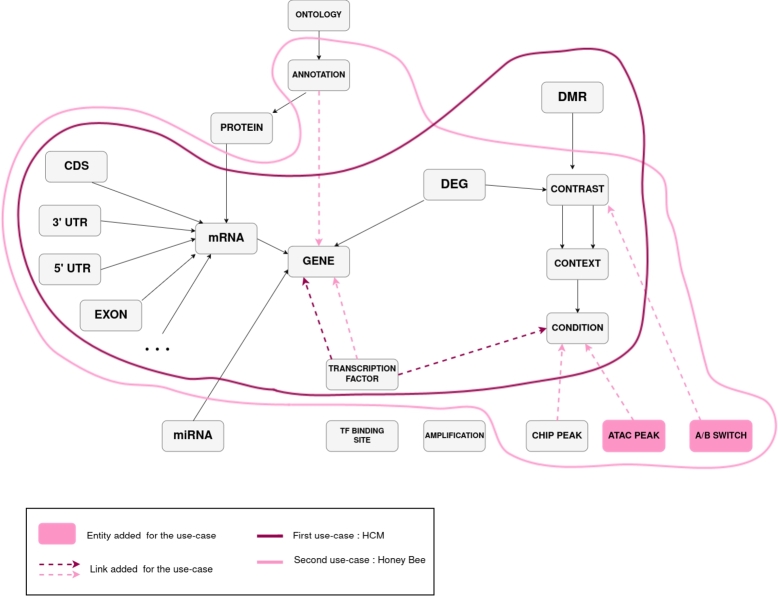
**Use-case mappings to the integration schema.** The entities circled in dark pink are those populated in the first use-case, while those circled in light pink are populated in the second one. The colored boxes are additional entities extending the data schema in one use-case, while colored arrows are additional links. The first use-case easily aligns with the integration schema. The second one needed to add two entities.

As illustrated in Fig. 4, our data schema aligns well with the experimental plan of the authors. Because the experimental plan in [38] is limited to gene differential expression, differential methylation and annotation of transcription factor targets, only a subset of the full integration schema is required. The transcription factor target annotations were not described by their location on the genome. We were thus unable to link them to the data schema using FALDO relations but added two links to connect them to Genes and Conditions (dashed arrows in Fig. 4).

The data schema was populated with the GFF of the human genome and the results of [38] on an Askomics instance at https://hcm.askomics.org/. While populating the data schema with the genome annotation might take a few hours (3 h), integrating the different results file only takes a few seconds. Altogether, these data represent 1,289 GB, 81,684,450 RDF triples, 9,856,785 entities, 57 classes, 179 properties and 12 graphs.

### 3.3 Querying the data schema for biomedical data about HCM (use-case 1)

We used the Askomics tool to query the data. Askomics enables to build a query graphically by clicking on the various entities and applying constraints on their attributes. The corresponding SPARQL query is generated automatically, and can be saved and re-run. Using Askomics is not mandatory to query the schema. It would also have been possible to query the RDF database directly *via* SPARQL without using Askomics.

To reproduce the major results of [38], we had to query our data schema with eight different queries. All these queries are included in File S1 and saved in the dedicated Askomics instance (https://hcm.askomics.org/).

We found the exact same number of up- and down-regulated DEGs (691 and 835, respectively), and up- and down-regulated lncRNAs (264 and 207, respectively).

When analyzing co-regulated genes, we identified the same number of co-up-regulated genes (297), but surprisingly noticed a small difference in co-down-regulated genes (524 indicated in the paper *vs.* 523 in our query). We were able to relate this -1 difference to a small reporting error in [38]: their supplementary file includes 523 entries, even though 524 are stated in the main text.

We then reproduced the result that there are 1453 hyper-methylated and 3600 hypo-methylated DMRs in HCM *vs.* Control.

**Fig. 5:**
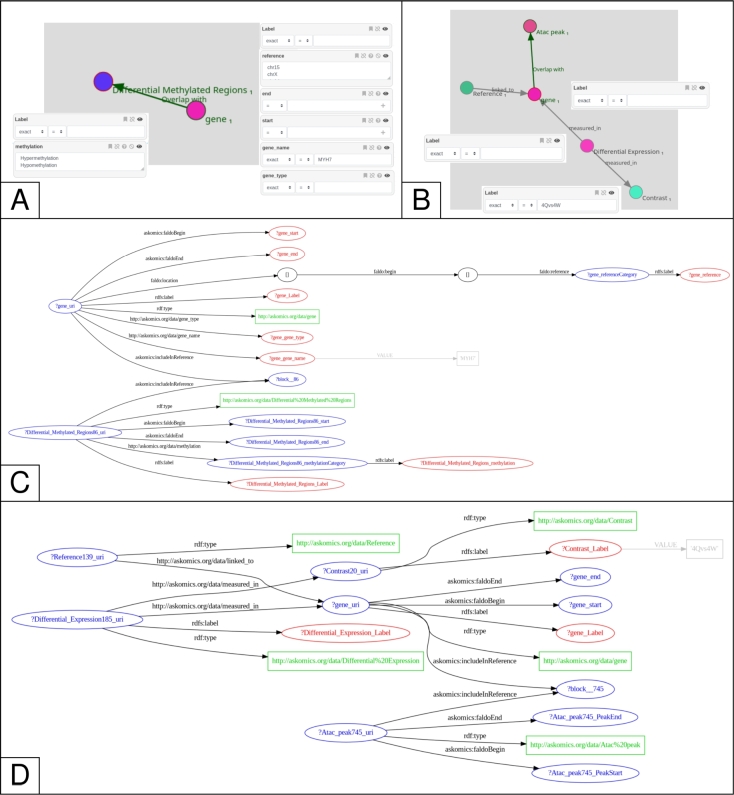
**Queries. (A)** Query creation on the HCM use-case with the Askomics tool: gene information for gene called MYH7, including whether it is associated with hypo- or hypermethylated regions. **(B)** Query creation on the honeybee use-case with the Askomics tool: number of genes involved in cast differentiation, that are differentially expressed in the 4Qvs4W contrast, and contain at least one ATAC-Seq peak. **(C)** Representation of the SPARQL query corresponding to panel A. **(D)** Representation of the SPARQL query corresponding to panel B.

We were unable to reproduce some results presented in the Figure 4 of [38] panel A, that focus on the expression level of genes in Controls, HCM and Fetuses. Some genes illustrated in this figure and mentioned as being key genes are actually not present in the differential expression tables of the corresponding supplementary data (supplementary table S3 in [38]). For example, MYH6 and MYH7 are not listed in the gene_name column of the supplementary table S3, and neither are their corresponding gene_id. This might be due to a small bug in the differential expression supplementary tables of [38]. However, these genes can be present in other tables, for example MYH7 is associated with hyper-methylated regions in HCM *vs.* Controls (supplementary table S4 of [38]). In this scase, we are able to found this information using our data schema, as illustrated in the Fig. 5 A & 5 C.

### 3.4 Populating the data schema for environmental data about honeybee (use-case 2)

The second use-case concerns insects, and is extracted from “The diverging epigenomic landscapes of honeybee queens and workers revealed by multi-omic sequencing” by Zhang et al. [39]. It aims to better understand the mechanisms implied in honeybee cast differentiation at the epigenetic level.

The experimental plan of this study involves cohorts of queen (Q) or worker (W) bees, studied at 2 or 4 days. Two Contrasts are studied: Q *vs.* W at 2 days (2Qvs2W) and Q *vs.* W at 4 days (4Qvs4W). For each Contrast, the authors identified differentially expressed genes (DEGs) from RNA-Seq, and predictions of conformation switch (A → B or B → A) from Hi-C analysis. The data also contains chromatin accessibility information with ATAC-Seq peaks and ChIP-Seq peaks, which were provided in four tables for the four Conditions: 2Q, 2W, 4Q, and 4W.

This second use case is a little further away from our integration schema. It overlaps with the first use-case, apart from differential methylation, which we don't have here. Furthermore, the second use-case contains additional data about annotations, ChIP peaks, ATAC peaks and Hi-C results. The latter two needed to be added to the schema and connected.

The data schema was first populated with the GFF of the honeybee genome on a dedicated Askomics instance: https://honeybee.askomics.org/. Then the results of [39] available as supplementary tables were added. These data altogether represent 162,979 MB, 14,880,684 RDF triples, 2,064,192 entities, 74 classes, 233 properties and 17 graphs. The integration of the bee genome took half an hour, whereas the results files required only a few seconds.

### 3.5 Querying the data schema for environmental data about honeybee (use-case 2)

We used once again the Askomics tool to query the data. Overall, 36 queries were created to replicate the results of the article. All these queries are shown in file S2 and saved on the Askomics instance (https://honeybee.askomics.org/). We briefly summarize the results reproduced, that required to integrate results from various single -omics analysis.

*Hi-C*  There are more A/B switched regions identified in the 4Qvs4W Constrast than in the 2Qvs2W one. Concerning genes overlapping with these switches, 325 and 247 genes switched between conformations A/B in the 4Qvs4W and 2Qvs2W Constrasts, respectively.

*ATAC-Seq*  There are 253, 382, 4618 and 448 unique ATAC-Seq peaks in the 2Q, 2W, 4Q and 4W Conditions, respectively.

*DEGs + ATAC-Seq*  The 4Qvs4W Constrast yielded more differentially expressed genes overlapping unique ATAC-Seq peaks than the 2Qvs2W Constrast.

*DEGs + ATAC-Seq + manual annotations*  The 4Qvs4W Constrast yielded more differentially expressed genes overlapping unique ATAC-Seq peaks known to be involved in cast differentiation. One query counting these events is illustrated in Fig. 5 B & 5 D.

*ChIP-Seq*  Concerning ChIP-Seq, there are 37, 181, 703 and 578 unique ChIP-Seq peaks in the 2Q, 2W, 4Q, and 4W Conditions, respectively. Thus, there are more peaks identified in the 4 day Conditions both for Queen (Q) and for Workers (W). Regarless of the time-point studied, unique ChIP-Seq peaks are more abundant for Queens (Q) than for Workers (W).

*DEGs + ChIP-Seq*  The 4Qvs4W Constrast yielded more differentially expressed genes overlapping unique ChIP-Seq peaks than the 2Qvs2W Constrast.

*DEGs + ChIP-Seq + manual annotations*  The 4Qvs4W Constrast yielded more differentially expressed genes overlapping unique ChIP-Seq peaks known to be involved in cast differentiation.

*DEGs + Hi-C + ATAC-seq + ChIP*  The genes differentially expressed in the 4Qvs4W Contrast were more often linked to more than one epigenetic change, including Hi-C, ATAC-Seq and ChIP-Seq than found in the 2Qvs2W Contrast.

Overall, the conclusions of the study presented in [39] were reproduced with our queries.

### 3.6 Integration schema extensibility

The schema we developed aims to be generic but as we saw, it does not cover exactly every case. However it is extensible. For the first use-case, we added two links; for the second, we added two entities and some links. These are easy transformations as long as we have information to relate to another entity (to create direct links) or positions on the genome (to link via the FALDO ontology).

To go further in the integration and the queries on our schema, we extended it to a federated ontology. In the second use case, we had information linking genes to articles. From this table, we searched the DOIs of each article and created a SPARQL query (file S3) to obtain the corresponding SemOpenAlex IDs. SemOpenAlex^5^ is an ontology about scientific publications. With the updated Reference table and a SemOpenAlex abstraction file that we created (file S20), we were able to create a link between our data and the federated database of SemOpenAlex. This enabled us to search more information about the papers where these genes were identified as involved in the bee cast differentiation.

## 4 Discussion

This work aimed to propose a versatile schema for the late integration and querying of different types of -omics data. We focused on studies combining at least transcriptomic and epigenomic analyses. We chose to follow the Semantic Web principles because of their advantages: being extensible to any type of data, being able to reason on knowledge ontologies, and being easy to connect to external resources presented in RDF. We proposed an integration schema following these principles and connected to various ontologies, and show how this schema can be linked to external knowledge presented in RDF format. Given Life Science long history in developing dedicated ontologies (Gene Ontology, DisGeNET,^6^ HPO^7^...) and knowledge bases using RDF (UniProtKB,^8^ CheBI^9^...), the Semantic Web approach is particularly relevant. An additional benefit of storing data in a generic RDF database is the data's persistence and accessibility to a wide audience.

We chose Askomics for its capability to query RDF datasets *via* a graphical interface, eliminating the need to be familiar with SPARQL. Furthermore, this tool makes it possible to combine your own local datasets with public datasets.

Following the example of Ortho_KB or Pantools, recent efforts have been made to offer knowledge bases that can integrate and query several types of -omics data. Although effective and robust, thanks to the neo4J graph database management system, they do not benefit from the advantages of Semantic Web technologies, like the ability to reason easily on ontologies or to federate queries with external resources. Thus, to the best of our knowledge, our approach does not align with existing database models, and we are therefore not in a position to directly compare our results.

To validate our integration schema and highlight its genericity, we choose to reproduce two recently published studies in very different research fields: one in biomedicine and the other in environmental science. These two published studies included transcriptomic and epigenomic data, but each study also integrated distinct additional data, thus also questioning the flexibility of the integration schema. We populated the integration schema with data from the two use-cases and proved the schema was generic enough to integrate data from these two studies and was flexible enough to integrate the different additional resources of each study. We demonstrated the integration schema does not need to be entirely populated and is easily extensible and flexible. This schema also enabled to query the entities based on their position on the genome, thanks to the FALDO ontology.

One of the reasons for developing a generic schema for multi-omics data integration was to limit the use of little-reused *ad-hoc* integration scripts. We did, however, have to develop *ad-hoc* scripts to populate our integration schema with the use-case data. This is mainly due to the fact that we didn't work with the direct output of the analyses, but with tables extracted from published articles. We noticed these tables were not the direct output of the transcriptomics and epigenomics analysis, but were post-processed by authors to improve the readability of their supplementary tables. More precisely, results from one single analysis were presented in different spreadsheets depending on the contrast studied, or depending on the regulation sense (up-regulated or down-regulated). This led us to develop scripts to concatenate back these tables. These scripts will not be required when working on the direct outputs of the different -omics analysis.

As we have focused on two use case studies, we have not sought to investigate the scalability of our framework. We did, however, encounter no problems in populating our integration schema with our two use-cases, which contained over 81 million triples for the biomedical use-case, and over 15 million triples for the environmental use-case. To query this model, all queries including the more complex ones execute in less than five seconds. The integration schema thus seems to scale to classical multi-omics studies.

We were able to produce several queries in order to reproduce efficiently the published analyses, and validate the approach. But, as we were not experts of these studies, we did not attempt to test new biological hypotheses using the integration schema, although this schema may allow it.

**Fig. 6:**
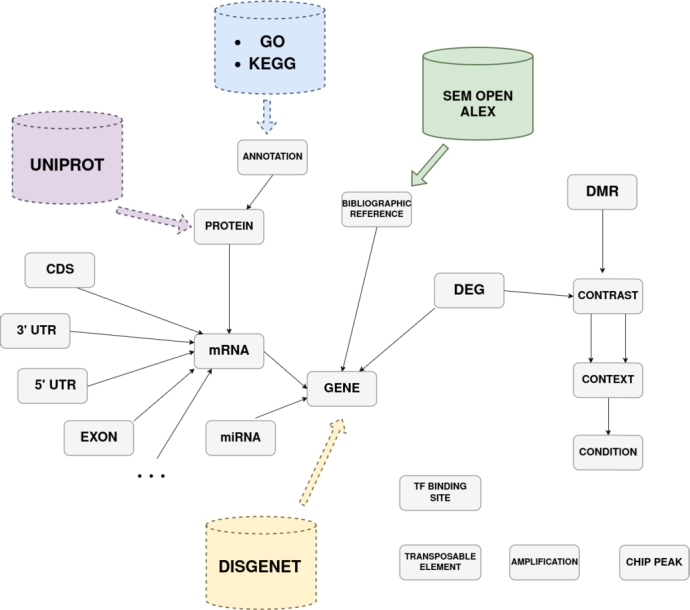
**Extension of the integration schema.** The integration schema can be extended to ontologies, allowing us to query these knowledge bases with our data. The SemOpenAlex database has been connected to the second use-case schema (represented with plain lines and arrows). Depending on the biological question to explore, several other databases such as UniProt, GO, KEGG or DisGeNET can be linked (represented with dashed lines and arrows).

As demonstrated with resources like SemOpenAlex, Gene Ontology, and KEGG, it is possible to create links to other external resources (see Fig. 6) and enable reasoning over the integrated data, with federated or local queries. For instance, proteins can be mapped to UniProtKB^10^ using their UniProt identifiers, or genes can be linked to the DisGeNET ontology^11^ to provide complementary information about their involvement in diseases. Additionally, the schema's high extensibility supports the rapid integration of other data types, such as those from proteomics or metabolomics studies. With the growth of multi-omics datasets, this approach offers a promising path for late data integration.

## CRediT authorship contribution statement

**Yael Tirlet:** Writing – review & editing, Writing – original draft, Formal analysis. **Matéo Boudet:** Writing – review & editing, Writing – original draft. **Emmanuelle Becker:** Writing – review & editing, Writing – original draft, Supervision, Conceptualization. **Fabrice Legeai:** Writing – review & editing, Writing – original draft, Supervision. **Olivier Dameron:** Writing – review & editing, Writing – original draft, Supervision, Conceptualization.

## Declaration of Competing Interest

The authors have no conflict of interest to disclose regarding this study.

## Appendix A Supplementary material

MMC 1 SPARQL queries of the HCM dataset. MMC 1

MMC 2 SPARQL queries of the Honeybee dataset. MMC 2

MMC 3 SPARQL query to retrieve SemOpenAlex identifiers. MMC 3

MMC 4 Final tables for HCM dataset integration. MMC 4

MMC 5 Final tables for Honeybee dataset integration. MMC 5

MMC 6 SemOpenAlex abstraction to query the ontology via Askomics for Honeybee use-case. MMC 6

## Footnotes

1 https://github.com/The-Sequence-Ontology/Specifications/blob/master/GFF3.md.

2 https://geneontology.org/.

3 https://www.genome.jp/kegg/.

4 https://askomics.org/.

5 https://semopenalex.org.

6 https://disgenet.com/.

7 https://hpo.jax.org/.

8 https://www.uniprot.org/.

9 https://www.ebi.ac.uk/chebi/.

10 https://www.uniprot.org/.

11 https://disgenet.com/.
