# Supplementary material for: Generic and queryable data integration schema for transcriptomics and epigenomics studies
Source: Comput Struct Biotechnol J. 2024 Nov 19;23:4232–41. doi: 10.1016/j.csbj.2024.11.022 (PMC11629147; doi:10.1016/j.csbj.2024.11.022)
Supplement: MMC 1 — SPARQL queries of the HCM dataset. [file mmc1.pdf]

HCM SPARQL Queries

PREFIXES :

```
PREFIX : <http://askomics.org/data/>
PREFIX askomics: <http://askomics.org/internal/>
PREFIX dc: <http://purl.org/dc/elements/1.1/>
PREFIX dcat: <http://www.w3.org/ns/dcat#>
PREFIX faldo: <http://biohackathon.org/resource/faldo/>
PREFIX owl: <http://www.w3.org/2002/07/owl#>
PREFIX prov: <http://www.w3.org/ns/prov#>
PREFIX rdf: <http://www.w3.org/1999/02/22-rdf-syntax-ns#>
PREFIX rdfs: <http://www.w3.org/2000/01/rdf-schema#>
PREFIX skos: <http://www.w3.org/2004/02/skos/core#>
PREFIX xsd: <http://www.w3.org/2001/XMLSchema#>
```

1- Number of Down-regulated lncRNA

```
SELECT DISTINCT ?Differential_Expression1_Label
WHERE {
   ?Differential_Expression1_uri rdf:type <http://askomics.org/data/Differential%20Expression> .
   ?Differential_Expression1_uri rdfs:label ?Differential_Expression1_Label .
   ?Differential_Expression1_uri <http://askomics.org/data/Type> ?
Differential_Expression1_TypeCategory .
   ?Differential_Expression1_uri <http://askomics.org/data/Expression> ?
Differential_Expression1_ExpressionCategory .
   VALUES ?Differential_Expression1_TypeCategory { <http://askomics.org/data/lncRNA
%20expression> }
   VALUES ?Differential_Expression1_ExpressionCategory { <http://askomics.org/data/DOWN> }
}
```

2- Number of Up-regulated lncRNA

```
SELECT DISTINCT ?Differential_Expression1_Label
WHERE {
   ?Differential_Expression1_uri rdf:type <http://askomics.org/data/Differential%20Expression> .
   ?Differential_Expression1_uri rdfs:label ?Differential_Expression1_Label .
   ?Differential_Expression1_uri <http://askomics.org/data/Type> ?
Differential_Expression1_TypeCategory .
   ?Differential_Expression1_uri <http://askomics.org/data/Expression> ?
Differential_Expression1_ExpressionCategory .
   VALUES ?Differential_Expression1_TypeCategory { <http://askomics.org/data/lncRNA
%20expression> }
   VALUES ?Differential_Expression1_ExpressionCategory { <http://askomics.org/data/UP> }
}
```

3- Number of hypomethylated DMRs

```
SELECT DISTINCT ?Differential_Methylated_Regions1_Label
WHERE {
    ?Differential_Methylated_Regions1_uri rdf:type <http://askomics.org/data/Differential
%20Methylated%20Regions> .
    ?Differential_Methylated_Regions1_uri rdfs:label ?Differential_Methylated_Regions1_Label .
    ?Differential_Methylated_Regions1_uri <http://askomics.org/data/methylation> ?
Differential_Methylated_Regions1_methylationCategory .
    VALUES ?Differential_Methylated_Regions1_methylationCategory {
<http://askomics.org/data/Hypomethylation> }
}
```

4- Number of hypermethylated DMRs

```
SELECT DISTINCT ?Differential_Methylated_Regions1_Label
WHERE {
    ?Differential_Methylated_Regions1_uri rdf:type <http://askomics.org/data/Differential
%20Methylated%20Regions> .
    ?Differential_Methylated_Regions1_uri rdfs:label ?Differential_Methylated_Regions1_Label .
    ?Differential_Methylated_Regions1_uri <http://askomics.org/data/methylation> ?
Differential_Methylated_Regions1_methylationCategory .
    VALUES ?Differential_Methylated_Regions1_methylationCategory {
<http://askomics.org/data/Hypermethylation> }
}
```

5- Number of Up-regulated DEGs

```
SELECT DISTINCT ?Differential_Expression1_Label ?Differential_Expression1_Expression
WHERE {
    ?Differential_Expression1_uri rdf:type <http://askomics.org/data/Differential%20Expression> .
    ?Differential_Expression1_uri rdfs:label ?Differential_Expression1_Label .
    ?Differential_Expression1_uri <http://askomics.org/data/Type> ?
Differential_Expression1_TypeCategory .
    ?Differential_Expression1_uri <http://askomics.org/data/Expression> ?
Differential_Expression1_ExpressionCategory .
    ?Differential_Expression1_ExpressionCategory rdfs:label ?
Differential_Expression1_Expression .
    VALUES ?Differential_Expression1_TypeCategory { <http://askomics.org/data/Gene
%20expression> }
    VALUES ?Differential_Expression1_ExpressionCategory { <http://askomics.org/data/UP> }
}
```

6- Number of Down-regulated DEGs

```
SELECT DISTINCT ?Differential_Expression1_Label ?Differential_Expression1_Expression
WHERE {
   ?Differential_Expression1_uri rdf:type <http://askomics.org/data/Differential%20Expression> .
   ?Differential_Expression1_uri rdfs:label ?Differential_Expression1_Label .
   ?Differential_Expression1_uri <http://askomics.org/data/Type> ?
Differential_Expression1_TypeCategory .
   ?Differential_Expression1_uri <http://askomics.org/data/Expression> ?
Differential_Expression1_ExpressionCategory .
   ?Differential_Expression1_ExpressionCategory rdfs:label ?
Differential_Expression1_Expression .
   VALUES ?Differential_Expression1_TypeCategory { <http://askomics.org/data/Gene
%20expression> }
   VALUES ?Differential_Expression1_ExpressionCategory { <http://askomics.org/data/DOWN> }
}
```

7- Number of co-Up-regulated DEGs

```
SELECT DISTINCT ?Differential_Expression1_Label ?Differential_Expression1_Expression
WHERE {
   ?Differential_Expression1_uri rdf:type <http://askomics.org/data/Differential%20Expression> .
   ?Differential_Expression1_uri rdfs:label ?Differential_Expression1_Label .
   ?Differential_Expression1_uri <http://askomics.org/data/Type> ?
Differential_Expression1_TypeCategory .
   ?Differential_Expression1_uri <http://askomics.org/data/Expression> ?
Differential_Expression1_ExpressionCategory .
   ?Differential_Expression1_ExpressionCategory rdfs:label ?
Differential_Expression1_Expression .
   VALUES ?Differential_Expression1_TypeCategory { <http://askomics.org/data/Gene%20Co-
expression> }
   VALUES ?Differential_Expression1_ExpressionCategory { <http://askomics.org/data/UP> }
}
```

8- Number of co-Down-regulated DEGs

```
SELECT DISTINCT ?Differential_Expression1_Label ?Differential_Expression1_Expression
WHERE {
   ?Differential_Expression1_uri rdf:type <http://askomics.org/data/Differential%20Expression> .
   ?Differential_Expression1_uri rdfs:label ?Differential_Expression1_Label .
   ?Differential_Expression1_uri <http://askomics.org/data/Type> ?
Differential_Expression1_TypeCategory .
   ?Differential_Expression1_uri <http://askomics.org/data/Expression> ?
Differential_Expression1_ExpressionCategory .
   ?Differential_Expression1_ExpressionCategory rdfs:label ?
Differential_Expression1_Expression .
   VALUES ?Differential_Expression1_TypeCategory { <http://askomics.org/data/Gene%20Co-
expression> }
   VALUES ?Differential_Expression1_ExpressionCategory { <http://askomics.org/data/DOWN> }
}
```
