# Supplementary material for: Generic and queryable data integration schema for transcriptomics and epigenomics studies
Source: Comput Struct Biotechnol J. 2024 Nov 19;23:4232–41. doi: 10.1016/j.csbj.2024.11.022 (PMC11629147; doi:10.1016/j.csbj.2024.11.022)
Supplement: MMC 2 — SPARQL queries of the Honeybee dataset. [file mmc2.pdf]

HoneyBee SPARQL Queries

PREFIXES :

```
PREFIX : <http://askomics.org/data/>
PREFIX askomics: <http://askomics.org/internal/>
PREFIX dc: <http://purl.org/dc/elements/1.1/>
PREFIX dcat: <http://www.w3.org/ns/dcat#>
PREFIX faldo: <http://biohackathon.org/resource/faldo/>
PREFIX owl: <http://www.w3.org/2002/07/owl#>
PREFIX prov: <http://www.w3.org/ns/prov#>
PREFIX rdf: <http://www.w3.org/1999/02/22-rdf-syntax-ns#>
PREFIX rdfs: <http://www.w3.org/2000/01/rdf-schema#>
PREFIX skos: <http://www.w3.org/2004/02/skos/core#>
PREFIX xsd: <http://www.w3.org/2001/XMLSchema#>
```

1- Number of Switch in the Contrast 2Qvs2W

```
SELECT DISTINCT ?Switch_A_B1_Label ?Switch_A_B1_direction ?Contrast1_Label
WHERE {
    ?Switch_A_B1_uri <http://askomics.org/data/measured_in> ?Contrast53_uri .
    ?Switch_A_B1_uri rdf:type <http://askomics.org/data/Switch%20A/B> .
    ?Switch_A_B1_uri rdfs:label ?Switch_A_B1_Label .
    ?Switch_A_B1_uri <http://askomics.org/data/direction> ?Switch_A_B1_directionCategory .
    ?Switch_A_B1_directionCategory rdfs:label ?Switch_A_B1_direction .
    ?Contrast53_uri rdf:type <http://askomics.org/data/Contrast> .
    ?Contrast53_uri rdfs:label ?Contrast1_Label .
    VALUES ?Contrast1_Label { '2Qvs2W' } .
}
```

2- Number of Switch in the Contrast 4Qvs4W

```
SELECT DISTINCT ?Switch_A_B1_Label ?Switch_A_B1_direction ?Contrast1_Label
WHERE {
    ?Switch_A_B1_uri <http://askomics.org/data/measured_in> ?Contrast53_uri .
    ?Switch_A_B1_uri rdf:type <http://askomics.org/data/Switch%20A/B> .
    ?Switch_A_B1_uri rdfs:label ?Switch_A_B1_Label .
    ?Switch_A_B1_uri <http://askomics.org/data/direction> ?Switch_A_B1_directionCategory .
    ?Switch_A_B1_directionCategory rdfs:label ?Switch_A_B1_direction .
    ?Contrast53_uri rdf:type <http://askomics.org/data/Contrast> .
    ?Contrast53_uri rdfs:label ?Contrast1_Label .
    VALUES ?Contrast1_Label { '4Qvs4W' } .
}
```

3- Number of Switch in the Contrast 2Qvs2W with gene overlap

```
SELECT DISTINCT ?Switch_A_B1_Label ?Switch_A_B1_direction ?Contrast1_Label ?
gene1_Label
WHERE {
   ?Switch_A_B1_uri <http://askomics.org/data/measured_in> ?Contrast53_uri .
   ?Switch_A_B1_uri askomics:includeInReference ?block_1_265 .
   ?gene265_uri askomics:includeInReference ?block_1_265 .
   ?Switch_A_B1_uri rdf:type <http://askomics.org/data/Switch%20A/B> .
   ?Switch_A_B1_uri rdfs:label ?Switch_A_B1_Label .
   ?Switch_A_B1_uri askomics:faldoEnd ?Switch_A_B1_binend .
   ?Switch_A_B1_uri askomics:faldoBegin ?Switch_A_B1_binstart .
   ?Switch_A_B1_uri <http://askomics.org/data/direction> ?Switch_A_B1_directionCategory .
   ?Switch_A_B1_directionCategory rdfs:label ?Switch_A_B1_direction .
   ?Contrast53_uri rdf:type <http://askomics.org/data/Contrast> .
   ?Contrast53_uri rdfs:label ?Contrast1_Label .
   ?gene265_uri rdf:type <http://askomics.org/data/gene> .
   ?gene265_uri rdfs:label ?gene1_Label .
   ?gene265_uri askomics:faldoBegin ?gene265_start .
   ?gene265_uri askomics:faldoEnd ?gene265_end .
   FILTER ((?gene265_start >= ?Switch_A_B1_binstart && ?gene265_start <= ?
Switch_A_B1_binend) || (?gene265_end >= ?Switch_A_B1_binstart && ?gene265_end <= ?
Switch_A_B1_binend) || (?Switch_A_B1_binstart >= ?gene265_start && ?Switch_A_B1_binend
<= ?gene265_end))
   VALUES ?Contrast1_Label { '2Qvs2W' } .
}
```

4- **Number of Switch in the Contrast 4Qvs4W with gene overlap**

```
SELECT DISTINCT ?Switch_A_B1_Label ?Switch_A_B1_direction ?Contrast1_Label ?
gene1_Label
WHERE {
   ?Switch_A_B1_uri <http://askomics.org/data/measured_in> ?Contrast53_uri .
   ?Switch_A_B1_uri askomics:includeInReference ?block_1_89 .
   ?gene89_uri askomics:includeInReference ?block_1_89 .
   ?Switch_A_B1_uri rdf:type <http://askomics.org/data/Switch%20A/B> .
   ?Switch_A_B1_uri rdfs:label ?Switch_A_B1_Label .
   ?Switch_A_B1_uri askomics:faldoEnd ?Switch_A_B1_binend .
   ?Switch_A_B1_uri askomics:faldoBegin ?Switch_A_B1_binstart .
   ?Switch_A_B1_uri <http://askomics.org/data/direction> ?Switch_A_B1_directionCategory .
   ?Switch_A_B1_directionCategory rdfs:label ?Switch_A_B1_direction .
   ?Contrast53_uri rdf:type <http://askomics.org/data/Contrast> .
   ?Contrast53_uri rdfs:label ?Contrast1_Label .
   ?gene89_uri rdf:type <http://askomics.org/data/gene> .
   ?gene89_uri rdfs:label ?gene1_Label .
   ?gene89_uri askomics:faldoBegin ?gene89_start .
   ?gene89_uri askomics:faldoEnd ?gene89_end .
   FILTER ((?gene89_start >= ?Switch_A_B1_binstart && ?gene89_start <= ?
Switch_A_B1_binend) || (?gene89_end >= ?Switch_A_B1_binstart && ?gene89_end <= ?
Switch_A_B1_binend) || (?Switch_A_B1_binstart >= ?gene89_start && ?Switch_A_B1_binend <=
?gene89_end))
   VALUES ?Contrast1_Label { '4Qvs4W' } .
}
```

5- Number of unique ATAC peaks in 2Q Condition

```
SELECT DISTINCT ?Atac_peak1_Label ?Condition1_Label ?Condition1_Days ?Condition1_Cast
WHERE {
    ?Atac_peak1_uri <http://askomics.org/data/measured_in> ?Condition57_uri .
    ?Atac_peak1_uri rdf:type <http://askomics.org/data/Atac%20peak> .
    ?Atac_peak1_uri rdfs:label ?Atac_peak1_Label .
    ?Condition57_uri rdf:type <http://askomics.org/data/Condition> .
    ?Condition57_uri rdfs:label ?Condition1_Label .
    ?Condition57_uri <http://askomics.org/data/Days> ?Condition57_DaysCategory .
    ?Condition57_DaysCategory rdfs:label ?Condition1_Days .
    ?Condition57_uri <http://askomics.org/data/Cast> ?Condition57_CastCategory .
    ?Condition57_CastCategory rdfs:label ?Condition1_Cast .
    VALUES ?Condition57_DaysCategory { <http://askomics.org/data/2> }
    VALUES ?Condition57_CastCategory { <http://askomics.org/data/Queen> }
}
```

6- Number of unique ATAC peaks in 2W Condition

```
SELECT DISTINCT ?Atac_peak1_Label ?Condition1_Label ?Condition1_Days ?Condition1_Cast
WHERE {
    ?Atac_peak1_uri <http://askomics.org/data/measured_in> ?Condition57_uri .
    ?Atac_peak1_uri rdf:type <http://askomics.org/data/Atac%20peak> .
    ?Atac_peak1_uri rdfs:label ?Atac_peak1_Label .
    ?Condition57_uri rdf:type <http://askomics.org/data/Condition> .
    ?Condition57_uri rdfs:label ?Condition1_Label .
    ?Condition57_uri <http://askomics.org/data/Days> ?Condition57_DaysCategory .
    ?Condition57_DaysCategory rdfs:label ?Condition1_Days .
    ?Condition57_uri <http://askomics.org/data/Cast> ?Condition57_CastCategory .
    ?Condition57_CastCategory rdfs:label ?Condition1_Cast .
    VALUES ?Condition57_DaysCategory { <http://askomics.org/data/2> }
    VALUES ?Condition57_CastCategory { <http://askomics.org/data/Worker> }
}
```

7- Number of unique ATAC peaks in 4Q Condition

```
SELECT DISTINCT ?Atac_peak1_Label ?Condition1_Label ?Condition1_Days ?Condition1_Cast
WHERE {
    ?Atac_peak1_uri <http://askomics.org/data/measured_in> ?Condition57_uri .
    ?Atac_peak1_uri rdf:type <http://askomics.org/data/Atac%20peak> .
    ?Atac_peak1_uri rdfs:label ?Atac_peak1_Label .
    ?Condition57_uri rdf:type <http://askomics.org/data/Condition> .
    ?Condition57_uri rdfs:label ?Condition1_Label .
    ?Condition57_uri <http://askomics.org/data/Days> ?Condition57_DaysCategory .
    ?Condition57_DaysCategory rdfs:label ?Condition1_Days .
    ?Condition57_uri <http://askomics.org/data/Cast> ?Condition57_CastCategory .
    ?Condition57_CastCategory rdfs:label ?Condition1_Cast .
    VALUES ?Condition57_DaysCategory { <http://askomics.org/data/4> }
    VALUES ?Condition57_CastCategory { <http://askomics.org/data/Queen> }
}
```

8- Number of unique ATAC peaks in 4W Condition

```
SELECT DISTINCT ?Atac_peak1_Label ?Condition1_Label ?Condition1_Days ?Condition1_Cast
WHERE {
    ?Atac_peak1_uri <http://askomics.org/data/measured_in> ?Condition57_uri .
    ?Atac_peak1_uri rdf:type <http://askomics.org/data/Atac%20peak> .
    ?Atac_peak1_uri rdfs:label ?Atac_peak1_Label .
    ?Condition57_uri rdf:type <http://askomics.org/data/Condition> .
    ?Condition57_uri rdfs:label ?Condition1_Label .
    ?Condition57_uri <http://askomics.org/data/Days> ?Condition57_DaysCategory .
    ?Condition57_DaysCategory rdfs:label ?Condition1_Days .
    ?Condition57_uri <http://askomics.org/data/Cast> ?Condition57_CastCategory .
    ?Condition57_CastCategory rdfs:label ?Condition1_Cast .
    VALUES ?Condition57_DaysCategory { <http://askomics.org/data/4> }
    VALUES ?Condition57_CastCategory { <http://askomics.org/data/Worker> }
}
```

9- Number of ATAC peaks that overlap with gene differentially expressed in Contrast 2Qvs2W

```
SELECT DISTINCT ?Atac_peak1_Label ?gene1_Label ?Differential_Expression1_Label ?
Contrast1_Label
WHERE {
    ?Atac_peak1_uri askomics:includeInReference ?block_1_91 .
    ?gene91_uri askomics:includeInReference ?block_1_91 .
    ?Differential_Expression333_uri <http://askomics.org/data/measured_in> ?gene91_uri .
    ?Differential_Expression333_uri <http://askomics.org/data/measured_in> ?Contrast349_uri .
    ?Atac_peak1_uri rdf:type <http://askomics.org/data/Atac%20peak> .
    ?Atac_peak1_uri rdfs:label ?Atac_peak1_Label .
    ?Atac_peak1_uri askomics:faldoEnd ?Atac_peak1_PeakEnd .
    ?Atac_peak1_uri askomics:faldoBegin ?Atac_peak1_PeakStart .
    ?gene91_uri rdf:type <http://askomics.org/data/gene> .
    ?gene91_uri rdfs:label ?gene1_Label .
    ?gene91_uri askomics:faldoBegin ?gene91_start .
    ?gene91_uri askomics:faldoEnd ?gene91_end .
    ?Differential_Expression333_uri rdf:type <http://askomics.org/data/Differential
%20Expression> .
    ?Differential_Expression333_uri rdfs:label ?Differential_Expression1_Label .
    ?Contrast349_uri rdf:type <http://askomics.org/data/Contrast> .
    ?Contrast349_uri rdfs:label ?Contrast1_Label .
    FILTER ((?gene91_start >= ?Atac_peak1_PeakStart && ?gene91_start <= ?
Atac_peak1_PeakEnd) || (?gene91_end >= ?Atac_peak1_PeakStart && ?gene91_end <= ?
Atac_peak1_PeakEnd) || (?Atac_peak1_PeakStart >= ?gene91_start && ?Atac_peak1_PeakEnd
<= ?gene91_end))
    VALUES ?Contrast1_Label { '2Qvs2W' } .
}
```

10- Number of ATAC peaks that overlap with gene differentially expressed in Contrast 4Qvs4W

```
SELECT DISTINCT ?Atac_peak1_Label ?gene1_Label ?Differential_Expression1_Label ?
Contrast1_Label
WHERE {
    ?Atac_peak1_uri askomics:includeInReference ?block_1_91 .
    ?gene91_uri askomics:includeInReference ?block_1_91 .
    ?Differential_Expression333_uri <http://askomics.org/data/measured_in> ?gene91_uri .
    ?Differential_Expression333_uri <http://askomics.org/data/measured_in> ?Contrast349_uri .
    ?Atac_peak1_uri rdf:type <http://askomics.org/data/Atac%20peak> .
    ?Atac_peak1_uri rdfs:label ?Atac_peak1_Label .
    ?Atac_peak1_uri askomics:faldoEnd ?Atac_peak1_PeakEnd .
    ?Atac_peak1_uri askomics:faldoBegin ?Atac_peak1_PeakStart .
    ?gene91_uri rdf:type <http://askomics.org/data/gene> .
    ?gene91_uri rdfs:label ?gene1_Label .
    ?gene91_uri askomics:faldoBegin ?gene91_start .
    ?gene91_uri askomics:faldoEnd ?gene91_end .
    ?Differential_Expression333_uri rdf:type <http://askomics.org/data/Differential
%20Expression> .
    ?Differential_Expression333_uri rdfs:label ?Differential_Expression1_Label .
    ?Contrast349_uri rdf:type <http://askomics.org/data/Contrast> .
    ?Contrast349_uri rdfs:label ?Contrast1_Label .
    FILTER ((?gene91_start >= ?Atac_peak1_PeakStart && ?gene91_start <= ?
Atac_peak1_PeakEnd) || (?gene91_end >= ?Atac_peak1_PeakStart && ?gene91_end <= ?
Atac_peak1_PeakEnd) || (?Atac_peak1_PeakStart >= ?gene91_start && ?Atac_peak1_PeakEnd
<= ?gene91_end))
    VALUES ?Contrast1_Label { '4Qvs4W' } .
}
```

11- Number of ATAC peaks that overlap with gene differentially expressed in Contrast 2Qvs2W that are implied in cast differentiation

```
SELECT DISTINCT ?Atac_peak1_Label ?gene1_Label ?Differential_Expression1_Label ?
Contrast1_Label ?Reference1_Label ?Reference1_Annotation
WHERE {
    ?Atac_peak1_uri askomics:includeInReference ?block_1_91 .
    ?gene91_uri askomics:includeInReference ?block_1_91 .
    ?Differential_Expression333_uri <http://askomics.org/data/measured_in> ?gene91_uri .
    ?Differential_Expression333_uri <http://askomics.org/data/measured_in> ?Contrast349_uri .
    ?Reference484_uri <http://askomics.org/data/linked_to> ?gene91_uri .
    ?Atac_peak1_uri rdf:type <http://askomics.org/data/Atac%20peak> .
    ?Atac_peak1_uri rdfs:label ?Atac_peak1_Label .
    ?Atac_peak1_uri askomics:faldoEnd ?Atac_peak1_PeakEnd .
    ?Atac_peak1_uri askomics:faldoBegin ?Atac_peak1_PeakStart .
    ?gene91_uri rdf:type <http://askomics.org/data/gene> .
    ?gene91_uri rdfs:label ?gene1_Label .
    ?gene91_uri askomics:faldoBegin ?gene91_start .
    ?gene91_uri askomics:faldoEnd ?gene91_end .
    ?Differential_Expression333_uri rdf:type <http://askomics.org/data/Differential
%20Expression> .
```

```
    ?Differential_Expression333_uri rdfs:label ?Differential_Expression1_Label .
    ?Contrast349_uri rdf:type <http://askomics.org/data/Contrast> .
    ?Contrast349_uri rdfs:label ?Contrast1_Label .
    ?Reference484_uri rdf:type <http://askomics.org/data/Reference> .
    ?Reference484_uri rdfs:label ?Reference1_Label .
    ?Reference484_uri <http://askomics.org/data/Annotation> ?Reference1_Annotation .
    FILTER ((?gene91_start >= ?Atac_peak1_PeakStart && ?gene91_start <= ?
Atac_peak1_PeakEnd) || (?gene91_end >= ?Atac_peak1_PeakStart && ?gene91_end <= ?
Atac_peak1_PeakEnd) || (?Atac_peak1_PeakStart >= ?gene91_start && ?Atac_peak1_PeakEnd
<= ?gene91_end))
    VALUES ?Contrast1_Label { '2Qvs2W' } .
}
```

12- Number of ATAC peaks that overlap with gene differentially expressed in Contrast 4Qvs4W that are implied in cast differentiation

```
SELECT DISTINCT ?Atac_peak1_Label ?gene1_Label ?Differential_Expression1_Label ?
Contrast1_Label ?Reference1_Label ?Reference1_Annotation
WHERE {
    ?Atac_peak1_uri askomics:includeInReference ?block_1_91 .
    ?gene91_uri askomics:includeInReference ?block_1_91 .
    ?Differential_Expression333_uri <http://askomics.org/data/measured_in> ?gene91_uri .
    ?Differential_Expression333_uri <http://askomics.org/data/measured_in> ?Contrast349_uri .
    ?Reference484_uri <http://askomics.org/data/linked_to> ?gene91_uri .
    ?Atac_peak1_uri rdf:type <http://askomics.org/data/Atac%20peak> .
    ?Atac_peak1_uri rdfs:label ?Atac_peak1_Label .
    ?Atac_peak1_uri askomics:faldoEnd ?Atac_peak1_PeakEnd .
    ?Atac_peak1_uri askomics:faldoBegin ?Atac_peak1_PeakStart .
    ?gene91_uri rdf:type <http://askomics.org/data/gene> .
    ?gene91_uri rdfs:label ?gene1_Label .
    ?gene91_uri askomics:faldoBegin ?gene91_start .
    ?gene91_uri askomics:faldoEnd ?gene91_end .
    ?Differential_Expression333_uri rdf:type <http://askomics.org/data/Differential
%20Expression> .
    ?Differential_Expression333_uri rdfs:label ?Differential_Expression1_Label .
    ?Contrast349_uri rdf:type <http://askomics.org/data/Contrast> .
    ?Contrast349_uri rdfs:label ?Contrast1_Label .
    ?Reference484_uri rdf:type <http://askomics.org/data/Reference> .
    ?Reference484_uri rdfs:label ?Reference1_Label .
    ?Reference484_uri <http://askomics.org/data/Annotation> ?Reference1_Annotation .
    FILTER ((?gene91_start >= ?Atac_peak1_PeakStart && ?gene91_start <= ?
Atac_peak1_PeakEnd) || (?gene91_end >= ?Atac_peak1_PeakStart && ?gene91_end <= ?
Atac_peak1_PeakEnd) || (?Atac_peak1_PeakStart >= ?gene91_start && ?Atac_peak1_PeakEnd
<= ?gene91_end))
    VALUES ?Contrast1_Label { '4Qvs4W' } .
}
```

13- Number of genes differentially expressed in 2Qvs2W that are implied in cast differentiation and contain unique ATAC peak(s)

```
SELECT DISTINCT ?gene1_Label ?Differential_Expression1_Label ?Contrast1_Label
WHERE {
    ?Reference139_uri <http://askomics.org/data/linked_to> ?gene1_uri .
    ?Differential_Expression185_uri <http://askomics.org/data/measured_in> ?gene1_uri .
    ?Differential_Expression185_uri <http://askomics.org/data/measured_in> ?Contrast201_uri .
    ?gene1_uri askomics:includeInReference ?block_1_745 .
    ?Atac_peak745_uri askomics:includeInReference ?block_1_745 .
    ?gene1_uri rdf:type <http://askomics.org/data/gene> .
    ?gene1_uri rdfs:label ?gene1_Label .
    ?gene1_uri askomics:faldoBegin ?gene1_start .
    ?gene1_uri askomics:faldoEnd ?gene1_end .
    ?Reference139_uri rdf:type <http://askomics.org/data/Reference> .
    ?Differential_Expression185_uri rdf:type <http://askomics.org/data/Differential
%20Expression> .
    ?Differential_Expression185_uri rdfs:label ?Differential_Expression1_Label .
    ?Contrast201_uri rdf:type <http://askomics.org/data/Contrast> .
    ?Contrast201_uri rdfs:label ?Contrast1_Label .
    ?Atac_peak745_uri rdf:type <http://askomics.org/data/Atac%20peak> .
    ?Atac_peak745_uri askomics:faldoEnd ?Atac_peak745_PeakEnd .
    ?Atac_peak745_uri askomics:faldoBegin ?Atac_peak745_PeakStart .
    FILTER ((?Atac_peak745_PeakStart >= ?gene1_start && ?Atac_peak745_PeakStart <= ?
gene1_end) || (?Atac_peak745_PeakEnd >= ?gene1_start && ?Atac_peak745_PeakEnd <= ?
gene1_end) || (?gene1_start >= ?Atac_peak745_PeakStart && ?gene1_end <= ?
Atac_peak745_PeakEnd))
    VALUES ?Contrast1_Label { '2Qvs2W' } .
}
```

14- Number of genes differentially expressed in 4Qvs4W that are implied in cast differentiation and contain unique ATAC peak(s)

```
SELECT DISTINCT ?gene1_Label ?Differential_Expression1_Label ?Contrast1_Label
WHERE {
    ?Reference139_uri <http://askomics.org/data/linked_to> ?gene1_uri .
    ?Differential_Expression185_uri <http://askomics.org/data/measured_in> ?gene1_uri .
    ?Differential_Expression185_uri <http://askomics.org/data/measured_in> ?Contrast201_uri .
    ?gene1_uri askomics:includeInReference ?block_1_745 .
    ?Atac_peak745_uri askomics:includeInReference ?block_1_745 .
    ?gene1_uri rdf:type <http://askomics.org/data/gene> .
    ?gene1_uri rdfs:label ?gene1_Label .
    ?gene1_uri askomics:faldoBegin ?gene1_start .
    ?gene1_uri askomics:faldoEnd ?gene1_end .
    ?Reference139_uri rdf:type <http://askomics.org/data/Reference> .
    ?Differential_Expression185_uri rdf:type <http://askomics.org/data/Differential
%20Expression> .
    ?Differential_Expression185_uri rdfs:label ?Differential_Expression1_Label .
    ?Contrast201_uri rdf:type <http://askomics.org/data/Contrast> .
    ?Contrast201_uri rdfs:label ?Contrast1_Label .
    ?Atac_peak745_uri rdf:type <http://askomics.org/data/Atac%20peak> .
```

```
    ?Atac_peak745_uri askomics:faldoEnd ?Atac_peak745_PeakEnd .
    ?Atac_peak745_uri askomics:faldoBegin ?Atac_peak745_PeakStart .
    FILTER ((?Atac_peak745_PeakStart >= ?gene1_start && ?Atac_peak745_PeakStart <= ?gene1_end) || (?Atac_peak745_PeakEnd >= ?gene1_start && ?Atac_peak745_PeakEnd <= ?gene1_end) || (?gene1_start >= ?Atac_peak745_PeakStart && ?gene1_end <= ?Atac_peak745_PeakEnd))
    VALUES ?Contrast1_Label { '4Qvs4W' } .
}
```

15- Number of unique Chip peaks in 2Q Condition

```
SELECT DISTINCT ?Chip_peak1_Label ?Condition1_Days ?Condition1_Cast
WHERE {
    ?Chip_peak1_uri <http://askomics.org/data/measured_in> ?Condition57_uri .
    ?Chip_peak1_uri rdf:type <http://askomics.org/data/Chip%20peak> .
    ?Chip_peak1_uri rdfs:label ?Chip_peak1_Label .
    ?Condition57_uri rdf:type <http://askomics.org/data/Condition> .
    ?Condition57_uri <http://askomics.org/data/Days> ?Condition57_DaysCategory .
    ?Condition57_DaysCategory rdfs:label ?Condition1_Days .
    ?Condition57_uri <http://askomics.org/data/Cast> ?Condition57_CastCategory .
    ?Condition57_CastCategory rdfs:label ?Condition1_Cast .
    VALUES ?Condition57_DaysCategory { <http://askomics.org/data/2> }
    VALUES ?Condition57_CastCategory { <http://askomics.org/data/Queen> }
}
```

16- Number of unique Chip peaks in 2W Condition

```
SELECT DISTINCT ?Chip_peak1_Label ?Condition1_Days ?Condition1_Cast
WHERE {
    ?Chip_peak1_uri <http://askomics.org/data/measured_in> ?Condition57_uri .
    ?Chip_peak1_uri rdf:type <http://askomics.org/data/Chip%20peak> .
    ?Chip_peak1_uri rdfs:label ?Chip_peak1_Label .
    ?Condition57_uri rdf:type <http://askomics.org/data/Condition> .
    ?Condition57_uri <http://askomics.org/data/Days> ?Condition57_DaysCategory .
    ?Condition57_DaysCategory rdfs:label ?Condition1_Days .
    ?Condition57_uri <http://askomics.org/data/Cast> ?Condition57_CastCategory .
    ?Condition57_CastCategory rdfs:label ?Condition1_Cast .
    VALUES ?Condition57_DaysCategory { <http://askomics.org/data/2> }
    VALUES ?Condition57_CastCategory { <http://askomics.org/data/Worker> }
}
```

17- Number of unique Chip peaks in 4Q Condition

```
SELECT DISTINCT ?Chip_peak1_Label ?Condition1_Days ?Condition1_Cast
WHERE {
    ?Chip_peak1_uri <http://askomics.org/data/measured_in> ?Condition57_uri .
    ?Chip_peak1_uri rdf:type <http://askomics.org/data/Chip%20peak> .
    ?Chip_peak1_uri rdfs:label ?Chip_peak1_Label .
    ?Condition57_uri rdf:type <http://askomics.org/data/Condition> .
    ?Condition57_uri <http://askomics.org/data/Days> ?Condition57_DaysCategory .
    ?Condition57_DaysCategory rdfs:label ?Condition1_Days .
    ?Condition57_uri <http://askomics.org/data/Cast> ?Condition57_CastCategory .
    ?Condition57_CastCategory rdfs:label ?Condition1_Cast .
    VALUES ?Condition57_DaysCategory { <http://askomics.org/data/4> }
    VALUES ?Condition57_CastCategory { <http://askomics.org/data/Queen> }
}
```

18- Number of unique Chip peaks in 4W Condition

```
SELECT DISTINCT ?Chip_peak1_Label ?Condition1_Days ?Condition1_Cast
WHERE {
    ?Chip_peak1_uri <http://askomics.org/data/measured_in> ?Condition57_uri .
    ?Chip_peak1_uri rdf:type <http://askomics.org/data/Chip%20peak> .
    ?Chip_peak1_uri rdfs:label ?Chip_peak1_Label .
    ?Condition57_uri rdf:type <http://askomics.org/data/Condition> .
    ?Condition57_uri <http://askomics.org/data/Days> ?Condition57_DaysCategory .
    ?Condition57_DaysCategory rdfs:label ?Condition1_Days .
    ?Condition57_uri <http://askomics.org/data/Cast> ?Condition57_CastCategory .
    ?Condition57_CastCategory rdfs:label ?Condition1_Cast .
    VALUES ?Condition57_DaysCategory { <http://askomics.org/data/4> }
    VALUES ?Condition57_CastCategory { <http://askomics.org/data/Worker> }
}
```

19- Number of unique Chip peaks that overlap with gene differentially expressed in Contrast 2Qvs2W

```
SELECT DISTINCT ?Chip_peak1_Label ?gene1_Label ?Differential_Expression1_Label ?
Differential_Expression1_Expression ?Contrast1_Label
WHERE {
    ?Chip_peak1_uri askomics:includeInReference ?block_1_31 .
    ?gene31_uri askomics:includeInReference ?block_1_31 .
    ?Differential_Expression273_uri <http://askomics.org/data/measured_in> ?gene31_uri .
    ?Differential_Expression273_uri <http://askomics.org/data/measured_in> ?Contrast293_uri .
    ?Chip_peak1_uri rdf:type <http://askomics.org/data/Chip%20peak> .
    ?Chip_peak1_uri rdfs:label ?Chip_peak1_Label .
    ?Chip_peak1_uri askomics:faldoBegin ?Chip_peak1_PeakStart .
    ?Chip_peak1_uri askomics:faldoEnd ?Chip_peak1_PeakEnd .
    ?gene31_uri rdf:type <http://askomics.org/data/gene> .
    ?gene31_uri rdfs:label ?gene1_Label .
    ?gene31_uri askomics:faldoBegin ?gene31_start .
```

```
    ?gene31_uri askomics:faldoEnd ?gene31_end .
    ?Differential_Expression273_uri rdf:type <http://askomics.org/data/Differential
%20Expression> .
    ?Differential_Expression273_uri rdfs:label ?Differential_Expression1_Label .
    ?Differential_Expression273_uri <http://askomics.org/data/Expression> ?
Differential_Expression273_ExpressionCategory .
    ?Differential_Expression273_ExpressionCategory rdfs:label ?
Differential_Expression1_Expression .
    ?Contrast293_uri rdf:type <http://askomics.org/data/Contrast> .
    ?Contrast293_uri rdfs:label ?Contrast1_Label .
    FILTER ((?gene31_start >= ?Chip_peak1_PeakStart && ?gene31_start <= ?
Chip_peak1_PeakEnd) || (?gene31_end >= ?Chip_peak1_PeakStart && ?gene31_end <= ?
Chip_peak1_PeakEnd) || (?Chip_peak1_PeakStart >= ?gene31_start && ?Chip_peak1_PeakEnd <=
?gene31_end))
    VALUES ?Contrast1_Label { '2Qvs2W' } .
}
```

20- Number of unique Chip peaks that overlap with gene differentially expressed in Contrast 4Qvs4W

```
SELECT DISTINCT ?Chip_peak1_Label ?gene1_Label ?Differential_Expression1_Label ?
Differential_Expression1_Expression ?Contrast1_Label
WHERE {
    ?Chip_peak1_uri askomics:includeInReference ?block_1_31 .
    ?gene31_uri askomics:includeInReference ?block_1_31 .
    ?Differential_Expression273_uri <http://askomics.org/data/measured_in> ?gene31_uri .
    ?Differential_Expression273_uri <http://askomics.org/data/measured_in> ?Contrast293_uri .
    ?Chip_peak1_uri rdf:type <http://askomics.org/data/Chip%20peak> .
    ?Chip_peak1_uri rdfs:label ?Chip_peak1_Label .
    ?Chip_peak1_uri askomics:faldoBegin ?Chip_peak1_PeakStart .
    ?Chip_peak1_uri askomics:faldoEnd ?Chip_peak1_PeakEnd .
    ?gene31_uri rdf:type <http://askomics.org/data/gene> .
    ?gene31_uri rdfs:label ?gene1_Label .
    ?gene31_uri askomics:faldoBegin ?gene31_start .
    ?gene31_uri askomics:faldoEnd ?gene31_end .
    ?Differential_Expression273_uri rdf:type <http://askomics.org/data/Differential
%20Expression> .
    ?Differential_Expression273_uri rdfs:label ?Differential_Expression1_Label .
    ?Differential_Expression273_uri <http://askomics.org/data/Expression> ?
Differential_Expression273_ExpressionCategory .
    ?Differential_Expression273_ExpressionCategory rdfs:label ?
Differential_Expression1_Expression .
    ?Contrast293_uri rdf:type <http://askomics.org/data/Contrast> .
    ?Contrast293_uri rdfs:label ?Contrast1_Label .
    FILTER ((?gene31_start >= ?Chip_peak1_PeakStart && ?gene31_start <= ?
Chip_peak1_PeakEnd) || (?gene31_end >= ?Chip_peak1_PeakStart && ?gene31_end <= ?
Chip_peak1_PeakEnd) || (?Chip_peak1_PeakStart >= ?gene31_start && ?Chip_peak1_PeakEnd <=
?gene31_end))
    VALUES ?Contrast1_Label { '4Qvs4W' } .
}
```

21- Number of Chip peaks that overlap with gene differentially expressed in Contrast 2Qvs2W that are implied in cast differentiation

```
SELECT DISTINCT ?Chip_peak1_Label ?gene1_Label ?Differential_Expression1_Label ?
Differential_Expression1_Expression ?Contrast1_Label ?Reference1_Label
WHERE {
    ?Chip_peak1_uri askomics:includeInReference ?block_1_31 .
    ?gene31_uri askomics:includeInReference ?block_1_31 .
    ?Differential_Expression273_uri <http://askomics.org/data/measured_in> ?gene31_uri .
    ?Differential_Expression273_uri <http://askomics.org/data/measured_in> ?Contrast293_uri .
    ?Reference428_uri <http://askomics.org/data/linked_to> ?gene31_uri .
    ?Chip_peak1_uri rdf:type <http://askomics.org/data/Chip%20peak> .
    ?Chip_peak1_uri rdfs:label ?Chip_peak1_Label .
    ?Chip_peak1_uri askomics:faldoBegin ?Chip_peak1_PeakStart .
    ?Chip_peak1_uri askomics:faldoEnd ?Chip_peak1_PeakEnd .
    ?gene31_uri rdf:type <http://askomics.org/data/gene> .
    ?gene31_uri rdfs:label ?gene1_Label .
    ?gene31_uri askomics:faldoBegin ?gene31_start .
    ?gene31_uri askomics:faldoEnd ?gene31_end .
    ?Differential_Expression273_uri rdf:type <http://askomics.org/data/Differential
%20Expression> .
    ?Differential_Expression273_uri rdfs:label ?Differential_Expression1_Label .
    ?Differential_Expression273_uri <http://askomics.org/data/Expression> ?
Differential_Expression273_ExpressionCategory .
    ?Differential_Expression273_ExpressionCategory rdfs:label ?
Differential_Expression1_Expression .
    ?Contrast293_uri rdf:type <http://askomics.org/data/Contrast> .
    ?Contrast293_uri rdfs:label ?Contrast1_Label .
    ?Reference428_uri rdf:type <http://askomics.org/data/Reference> .
    ?Reference428_uri rdfs:label ?Reference1_Label .
    FILTER ((?gene31_start >= ?Chip_peak1_PeakStart && ?gene31_start <= ?
Chip_peak1_PeakEnd) || (?gene31_end >= ?Chip_peak1_PeakStart && ?gene31_end <= ?
Chip_peak1_PeakEnd) || (?Chip_peak1_PeakStart >= ?gene31_start && ?Chip_peak1_PeakEnd <=
?gene31_end))
    VALUES ?Contrast1_Label { '2Qvs2W' } .
}
```

22- Number of Chip peaks that overlap with gene differentially expressed in Contrast 4Qvs4W that are implied in cast differentiation

```
SELECT DISTINCT ?Chip_peak1_Label ?gene1_Label ?Differential_Expression1_Label ?
Differential_Expression1_Expression ?Contrast1_Label ?Reference1_Label
WHERE {
    ?Chip_peak1_uri askomics:includeInReference ?block_1_31 .
    ?gene31_uri askomics:includeInReference ?block_1_31 .
    ?Differential_Expression273_uri <http://askomics.org/data/measured_in> ?gene31_uri .
    ?Differential_Expression273_uri <http://askomics.org/data/measured_in> ?Contrast293_uri .
    ?Reference428_uri <http://askomics.org/data/linked_to> ?gene31_uri .
    ?Chip_peak1_uri rdf:type <http://askomics.org/data/Chip%20peak> .
    ?Chip_peak1_uri rdfs:label ?Chip_peak1_Label .
    ?Chip_peak1_uri askomics:faldoBegin ?Chip_peak1_PeakStart .
```

```
    ?Chip_peak1_uri askomics:faldoEnd ?Chip_peak1_PeakEnd .
    ?gene31_uri rdf:type <http://askomics.org/data/gene> .
    ?gene31_uri rdfs:label ?gene1_Label .
    ?gene31_uri askomics:faldoBegin ?gene31_start .
    ?gene31_uri askomics:faldoEnd ?gene31_end .
    ?Differential_Expression273_uri rdf:type <http://askomics.org/data/Differential
%20Expression> .
    ?Differential_Expression273_uri rdfs:label ?Differential_Expression1_Label .
    ?Differential_Expression273_uri <http://askomics.org/data/Expression> ?
Differential_Expression273_ExpressionCategory .
    ?Differential_Expression273_ExpressionCategory rdfs:label ?
Differential_Expression1_Expression .
    ?Contrast293_uri rdf:type <http://askomics.org/data/Contrast> .
    ?Contrast293_uri rdfs:label ?Contrast1_Label .
    ?Reference428_uri rdf:type <http://askomics.org/data/Reference> .
    ?Reference428_uri rdfs:label ?Reference1_Label .
    FILTER ((?gene31_start >= ?Chip_peak1_PeakStart && ?gene31_start <= ?
Chip_peak1_PeakEnd) || (?gene31_end >= ?Chip_peak1_PeakStart && ?gene31_end <= ?
Chip_peak1_PeakEnd) || (?Chip_peak1_PeakStart >= ?gene31_start && ?Chip_peak1_PeakEnd <=
?gene31_end))
    VALUES ?Contrast1_Label { '4Qvs4W' } .
}
```

23- Number of genes differentially expressed in 2Qvs2W that are implied in cast differentiation and contain unique Chip peak(s)

```
SELECT DISTINCT ?gene1_Label
WHERE {
    ?Reference139_uri <http://askomics.org/data/linked_to> ?gene1_uri .
    ?Differential_Expression185_uri <http://askomics.org/data/measured_in> ?gene1_uri .
    ?Differential_Expression185_uri <http://askomics.org/data/measured_in> ?Contrast201_uri .
    ?gene1_uri askomics:includeInReference ?block_1_305 .
    ?Chip_peak305_uri askomics:includeInReference ?block_1_305 .
    ?gene1_uri rdf:type <http://askomics.org/data/gene> .
    ?gene1_uri rdfs:label ?gene1_Label .
    ?gene1_uri askomics:faldoBegin ?gene1_start .
    ?gene1_uri askomics:faldoEnd ?gene1_end .
    ?Reference139_uri rdf:type <http://askomics.org/data/Reference> .
    ?Differential_Expression185_uri rdf:type <http://askomics.org/data/Differential
%20Expression> .
    ?Contrast201_uri rdf:type <http://askomics.org/data/Contrast> .
    ?Contrast201_uri rdfs:label ?Contrast1_Label .
    ?Chip_peak305_uri rdf:type <http://askomics.org/data/Chip%20peak> .
    ?Chip_peak305_uri askomics:faldoBegin ?Chip_peak305_PeakStart .
    ?Chip_peak305_uri askomics:faldoEnd ?Chip_peak305_PeakEnd .
    FILTER ((?Chip_peak305_PeakStart >= ?gene1_start && ?Chip_peak305_PeakStart <= ?
gene1_end) || (?Chip_peak305_PeakEnd >= ?gene1_start && ?Chip_peak305_PeakEnd <= ?
gene1_end) || (?gene1_start >= ?Chip_peak305_PeakStart && ?gene1_end <= ?
Chip_peak305_PeakEnd))
    VALUES ?Contrast1_Label { '2Qvs2W' } .
}
```

24- Number of genes differentially expressed in 4Qvs4W that are implied in cast differentiation and contain unique Chip peak(s)

```
SELECT DISTINCT ?gene1_Label
WHERE {
    ?Reference139_uri <http://askomics.org/data/linked_to> ?gene1_uri .
    ?Differential_Expression185_uri <http://askomics.org/data/measured_in> ?gene1_uri .
    ?Differential_Expression185_uri <http://askomics.org/data/measured_in> ?Contrast201_uri .
    ?gene1_uri askomics:includeInReference ?block_1_305 .
    ?Chip_peak305_uri askomics:includeInReference ?block_1_305 .
    ?gene1_uri rdf:type <http://askomics.org/data/gene> .
    ?gene1_uri rdfs:label ?gene1_Label .
    ?gene1_uri askomics:faldoBegin ?gene1_start .
    ?gene1_uri askomics:faldoEnd ?gene1_end .
    ?Reference139_uri rdf:type <http://askomics.org/data/Reference> .
    ?Differential_Expression185_uri rdf:type <http://askomics.org/data/Differential
%20Expression> .
    ?Contrast201_uri rdf:type <http://askomics.org/data/Contrast> .
    ?Contrast201_uri rdfs:label ?Contrast1_Label .
    ?Chip_peak305_uri rdf:type <http://askomics.org/data/Chip%20peak> .
    ?Chip_peak305_uri askomics:faldoBegin ?Chip_peak305_PeakStart .
    ?Chip_peak305_uri askomics:faldoEnd ?Chip_peak305_PeakEnd .
    FILTER ((?Chip_peak305_PeakStart >= ?gene1_start && ?Chip_peak305_PeakStart <= ?
gene1_end) || (?Chip_peak305_PeakEnd >= ?gene1_start && ?Chip_peak305_PeakEnd <= ?
gene1_end) || (?gene1_start >= ?Chip_peak305_PeakStart && ?gene1_end <= ?
Chip_peak305_PeakEnd))
    VALUES ?Contrast1_Label { '4Qvs4W' } .
}
```

25- Number of genes differentially expressed in 2Qvs2W and contain unique ATAC peak(s)

```
SELECT DISTINCT ?gene1_Label ?Differential_Expression1_Label ?Contrast1_Label
WHERE {
    ?Differential_Expression185_uri <http://askomics.org/data/measured_in> ?gene1_uri .
    ?Differential_Expression185_uri <http://askomics.org/data/measured_in> ?Contrast201_uri .
    ?gene1_uri askomics:includeInReference ?block_1_745 .
    ?Atac_peak745_uri askomics:includeInReference ?block_1_745 .
    ?gene1_uri rdf:type <http://askomics.org/data/gene> .
    ?gene1_uri rdfs:label ?gene1_Label .
    ?gene1_uri askomics:faldoBegin ?gene1_start .
    ?gene1_uri askomics:faldoEnd ?gene1_end .
    ?Differential_Expression185_uri rdf:type <http://askomics.org/data/Differential
%20Expression> .
    ?Differential_Expression185_uri rdfs:label ?Differential_Expression1_Label .
    ?Contrast201_uri rdf:type <http://askomics.org/data/Contrast> .
    ?Contrast201_uri rdfs:label ?Contrast1_Label .
    ?Atac_peak745_uri rdf:type <http://askomics.org/data/Atac%20peak> .
    ?Atac_peak745_uri askomics:faldoEnd ?Atac_peak745_PeakEnd .
    ?Atac_peak745_uri askomics:faldoBegin ?Atac_peak745_PeakStart .
    FILTER ((?Atac_peak745_PeakStart >= ?gene1_start && ?Atac_peak745_PeakStart <= ?
gene1_end) || (?Atac_peak745_PeakEnd >= ?gene1_start && ?Atac_peak745_PeakEnd <= ?
gene1_end) || (?gene1_start >= ?Atac_peak745_PeakStart && ?gene1_end <= ?
Atac_peak745_PeakEnd))
    VALUES ?Contrast1_Label { '2Qvs2W' } . }
```

26- Number of gene differentially expressed in 4Qvs4W and contain unique ATAC peak(s)

```
SELECT DISTINCT ?gene1_Label ?Differential_Expression1_Label ?Contrast1_Label
WHERE {
   ?Differential_Expression185_uri <http://askomics.org/data/measured_in> ?gene1_uri .
   ?Differential_Expression185_uri <http://askomics.org/data/measured_in> ?Contrast201_uri .
   ?gene1_uri askomics:includeInReference ?block_1_745 .
   ?Atac_peak745_uri askomics:includeInReference ?block_1_745 .
   ?gene1_uri rdf:type <http://askomics.org/data/gene> .
   ?gene1_uri rdfs:label ?gene1_Label .
   ?gene1_uri askomics:faldoBegin ?gene1_start .
   ?gene1_uri askomics:faldoEnd ?gene1_end .
   ?Differential_Expression185_uri rdf:type <http://askomics.org/data/Differential
%20Expression> .
   ?Differential_Expression185_uri rdfs:label ?Differential_Expression1_Label .
   ?Contrast201_uri rdf:type <http://askomics.org/data/Contrast> .
   ?Contrast201_uri rdfs:label ?Contrast1_Label .
   ?Atac_peak745_uri rdf:type <http://askomics.org/data/Atac%20peak> .
   ?Atac_peak745_uri askomics:faldoEnd ?Atac_peak745_PeakEnd .
   ?Atac_peak745_uri askomics:faldoBegin ?Atac_peak745_PeakStart .
   FILTER ((?Atac_peak745_PeakStart >= ?gene1_start && ?Atac_peak745_PeakStart <= ?
gene1_end) || (?Atac_peak745_PeakEnd >= ?gene1_start && ?Atac_peak745_PeakEnd <= ?
gene1_end) || (?gene1_start >= ?Atac_peak745_PeakStart && ?gene1_end <= ?
Atac_peak745_PeakEnd))
   VALUES ?Contrast1_Label { '4Qvs4W' } .
}
```

27- Number of gene differentially expressed in 2Qvs2W and contain unique Chip peak(s)

```
SELECT DISTINCT ?gene1_Label
WHERE {
   ?Differential_Expression185_uri <http://askomics.org/data/measured_in> ?gene1_uri .
   ?Differential_Expression185_uri <http://askomics.org/data/measured_in> ?Contrast201_uri .
   ?gene1_uri askomics:includeInReference ?block_1_305 .
   ?Chip_peak305_uri askomics:includeInReference ?block_1_305 .
   ?gene1_uri rdf:type <http://askomics.org/data/gene> .
   ?gene1_uri rdfs:label ?gene1_Label .
   ?gene1_uri askomics:faldoBegin ?gene1_start .
   ?gene1_uri askomics:faldoEnd ?gene1_end .
   ?Differential_Expression185_uri rdf:type <http://askomics.org/data/Differential
%20Expression> .
   ?Contrast201_uri rdf:type <http://askomics.org/data/Contrast> .
   ?Contrast201_uri rdfs:label ?Contrast1_Label .
   ?Chip_peak305_uri rdf:type <http://askomics.org/data/Chip%20peak> .
   ?Chip_peak305_uri askomics:faldoBegin ?Chip_peak305_PeakStart .
   ?Chip_peak305_uri askomics:faldoEnd ?Chip_peak305_PeakEnd .
   FILTER ((?Chip_peak305_PeakStart >= ?gene1_start && ?Chip_peak305_PeakStart <= ?
gene1_end) || (?Chip_peak305_PeakEnd >= ?gene1_start && ?Chip_peak305_PeakEnd <= ?
gene1_end) || (?gene1_start >= ?Chip_peak305_PeakStart && ?gene1_end <= ?
Chip_peak305_PeakEnd))
   VALUES ?Contrast1_Label { '2Qvs2W' } .
}
```

28- Number of gene differentially expressed in 4Qvs4W and contain unique Chip peak(s)

```
SELECT DISTINCT ?gene1_Label
WHERE {
    ?Differential_Expression185_uri <http://askomics.org/data/measured_in> ?gene1_uri .
    ?Differential_Expression185_uri <http://askomics.org/data/measured_in> ?Contrast201_uri .
    ?gene1_uri askomics:includeInReference ?block_1_305 .
    ?Chip_peak305_uri askomics:includeInReference ?block_1_305 .
    ?gene1_uri rdf:type <http://askomics.org/data/gene> .
    ?gene1_uri rdfs:label ?gene1_Label .
    ?gene1_uri askomics:faldoBegin ?gene1_start .
    ?gene1_uri askomics:faldoEnd ?gene1_end .
    ?Differential_Expression185_uri rdf:type <http://askomics.org/data/Differential
%20Expression> .
    ?Contrast201_uri rdf:type <http://askomics.org/data/Contrast> .
    ?Contrast201_uri rdfs:label ?Contrast1_Label .
    ?Chip_peak305_uri rdf:type <http://askomics.org/data/Chip%20peak> .
    ?Chip_peak305_uri askomics:faldoBegin ?Chip_peak305_PeakStart .
    ?Chip_peak305_uri askomics:faldoEnd ?Chip_peak305_PeakEnd .
    FILTER ((?Chip_peak305_PeakStart >= ?gene1_start && ?Chip_peak305_PeakStart <= ?
gene1_end) || (?Chip_peak305_PeakEnd >= ?gene1_start && ?Chip_peak305_PeakEnd <= ?
gene1_end) || (?gene1_start >= ?Chip_peak305_PeakStart && ?gene1_end <= ?
Chip_peak305_PeakEnd))
    VALUES ?Contrast1_Label { '4Qvs4W' } .
}
```

29- Number of genes that are implied in cast differentiation, are differentially expressed in Contrast 2Qvs2W, contain unique Chip peak(s) and overlap with a Switch region

```
SELECT DISTINCT ?gene1_Label ?Differential_Expression1_Label ?Contrast1_Label ?
Contrast201_Label ?Condition1_Label
WHERE {
    ?Reference139_uri <http://askomics.org/data/linked_to> ?gene1_uri .
    ?Differential_Expression185_uri <http://askomics.org/data/measured_in> ?gene1_uri .
    ?Differential_Expression185_uri <http://askomics.org/data/measured_in> ?Contrast201_uri .
    ?gene1_uri askomics:includeInReference ?block_1_408 .
    ?Switch_A_B408_uri askomics:includeInReference ?block_1_408 .
    ?Switch_A_B408_uri <http://askomics.org/data/measured_in> ?Contrast542_uri .
    ?gene1_uri askomics:includeInReference ?block_1_607 .
    ?Chip_peak607_uri askomics:includeInReference ?block_1_607 .
    ?Chip_peak607_uri <http://askomics.org/data/measured_in> ?Condition710_uri .
    ?gene1_uri rdf:type <http://askomics.org/data/gene> .
    ?gene1_uri rdfs:label ?gene1_Label .
    ?gene1_uri askomics:faldoBegin ?gene1_start .
    ?gene1_uri askomics:faldoEnd ?gene1_end .
    ?Reference139_uri rdf:type <http://askomics.org/data/Reference> .
    ?Differential_Expression185_uri rdf:type <http://askomics.org/data/Differential
%20Expression> .
    ?Differential_Expression185_uri rdfs:label ?Differential_Expression1_Label .
    ?Contrast201_uri rdf:type <http://askomics.org/data/Contrast> .
    ?Contrast201_uri rdfs:label ?Contrast1_Label .
```

```
    ?Switch_A_B408_uri rdf:type <http://askomics.org/data/Switch%20A/B> .
    ?Switch_A_B408_uri askomics:faldoEnd ?Switch_A_B408_binend .
    ?Switch_A_B408_uri askomics:faldoBegin ?Switch_A_B408_binstart .
    ?Contrast542_uri rdf:type <http://askomics.org/data/Contrast> .
    ?Contrast542_uri rdfs:label ?Contrast201_Label .
    ?Chip_peak607_uri rdf:type <http://askomics.org/data/Chip%20peak> .
    ?Chip_peak607_uri askomics:faldoBegin ?Chip_peak607_PeakStart .
    ?Chip_peak607_uri askomics:faldoEnd ?Chip_peak607_PeakEnd .
    ?Condition710_uri rdf:type <http://askomics.org/data/Condition> .
    ?Condition710_uri rdfs:label ?Condition1_Label .
    ?Condition710_uri <http://askomics.org/data/Days> ?Condition710_DaysCategory .
    FILTER ((?Switch_A_B408_binstart >= ?gene1_start && ?Switch_A_B408_binstart <= ?
gene1_end) || (?Switch_A_B408_binend >= ?gene1_start && ?Switch_A_B408_binend <= ?
gene1_end) || (?gene1_start >= ?Switch_A_B408_binstart && ?gene1_end <= ?
Switch_A_B408_binend))
    FILTER ((?Chip_peak607_PeakStart >= ?gene1_start && ?Chip_peak607_PeakStart <= ?
gene1_end) || (?Chip_peak607_PeakEnd >= ?gene1_start && ?Chip_peak607_PeakEnd <= ?
gene1_end) || (?gene1_start >= ?Chip_peak607_PeakStart && ?gene1_end <= ?
Chip_peak607_PeakEnd))
    VALUES ?Contrast1_Label { '2Qvs2W' } .
    VALUES ?Condition710_DaysCategory { <http://askomics.org/data/2> }
}
```

30- Number of genes that are implied in cast differentiation, are differentially expressed in Contrast 4Qvs4W, contain unique Chip peak(s) and overlap with a Switch region

```
SELECT DISTINCT ?gene1_Label ?Differential_Expression1_Label ?Contrast1_Label ?
Contrast201_Label ?Condition1_Label
WHERE {
    ?Reference139_uri <http://askomics.org/data/linked_to> ?gene1_uri .
    ?Differential_Expression185_uri <http://askomics.org/data/measured_in> ?gene1_uri .
    ?Differential_Expression185_uri <http://askomics.org/data/measured_in> ?Contrast201_uri .
    ?gene1_uri askomics:includeInReference ?block_1_408 .
    ?Switch_A_B408_uri askomics:includeInReference ?block_1_408 .
    ?Switch_A_B408_uri <http://askomics.org/data/measured_in> ?Contrast542_uri .
    ?gene1_uri askomics:includeInReference ?block_1_607 .
    ?Chip_peak607_uri askomics:includeInReference ?block_1_607 .
    ?Chip_peak607_uri <http://askomics.org/data/measured_in> ?Condition710_uri .
    ?gene1_uri rdf:type <http://askomics.org/data/gene> .
    ?gene1_uri rdfs:label ?gene1_Label .
    ?gene1_uri askomics:faldoBegin ?gene1_start .
    ?gene1_uri askomics:faldoEnd ?gene1_end .
    ?Reference139_uri rdf:type <http://askomics.org/data/Reference> .
    ?Differential_Expression185_uri rdf:type <http://askomics.org/data/Differential
%20Expression> .
    ?Differential_Expression185_uri rdfs:label ?Differential_Expression1_Label .
    ?Contrast201_uri rdf:type <http://askomics.org/data/Contrast> .
    ?Contrast201_uri rdfs:label ?Contrast1_Label .
    ?Switch_A_B408_uri rdf:type <http://askomics.org/data/Switch%20A/B> .
    ?Switch_A_B408_uri askomics:faldoEnd ?Switch_A_B408_binend .
    ?Switch_A_B408_uri askomics:faldoBegin ?Switch_A_B408_binstart .
```

```
    ?Contrast542_uri rdf:type <http://askomics.org/data/Contrast> .
    ?Contrast542_uri rdfs:label ?Contrast201_Label .
    ?Chip_peak607_uri rdf:type <http://askomics.org/data/Chip%20peak> .
    ?Chip_peak607_uri askomics:faldoBegin ?Chip_peak607_PeakStart .
    ?Chip_peak607_uri askomics:faldoEnd ?Chip_peak607_PeakEnd .
    ?Condition710_uri rdf:type <http://askomics.org/data/Condition> .
    ?Condition710_uri rdfs:label ?Condition1_Label .
    ?Condition710_uri <http://askomics.org/data/Days> ?Condition710_DaysCategory .
    FILTER ((?Switch_A_B408_binstart >= ?gene1_start && ?Switch_A_B408_binstart <= ?
gene1_end) || (?Switch_A_B408_binend >= ?gene1_start && ?Switch_A_B408_binend <= ?
gene1_end) || (?gene1_start >= ?Switch_A_B408_binstart && ?gene1_end <= ?
Switch_A_B408_binend))
    FILTER ((?Chip_peak607_PeakStart >= ?gene1_start && ?Chip_peak607_PeakStart <= ?
gene1_end) || (?Chip_peak607_PeakEnd >= ?gene1_start && ?Chip_peak607_PeakEnd <= ?
gene1_end) || (?gene1_start >= ?Chip_peak607_PeakStart && ?gene1_end <= ?
Chip_peak607_PeakEnd))
    VALUES ?Contrast1_Label { '4Qvs4W' } .
    VALUES ?Condition710_DaysCategory { <http://askomics.org/data/4> }
}
```

31- Number of genes that are implied in cast differentiation, are differentially expressed in Contrast 2Qvs2W, contain unique ATAC peak(s) and overlap with a Switch region

```
SELECT DISTINCT ?gene1_Label ?Differential_Expression1_Label ?Contrast1_Label ?
Contrast201_Label
WHERE {
    ?Reference139_uri <http://askomics.org/data/linked_to> ?gene1_uri .
    ?Differential_Expression185_uri <http://askomics.org/data/measured_in> ?gene1_uri .
    ?Differential_Expression185_uri <http://askomics.org/data/measured_in> ?Contrast201_uri .
    ?gene1_uri askomics:includeInReference ?block_1_408 .
    ?Switch_A_B408_uri askomics:includeInReference ?block_1_408 .
    ?Switch_A_B408_uri <http://askomics.org/data/measured_in> ?Contrast542_uri .
    ?gene1_uri askomics:includeInReference ?block_1_847 .
    ?Atac_peak847_uri askomics:includeInReference ?block_1_847 .
    ?Atac_peak847_uri <http://askomics.org/data/measured_in> ?Condition948_uri .
    ?gene1_uri rdf:type <http://askomics.org/data/gene> .
    ?gene1_uri rdfs:label ?gene1_Label .
    ?gene1_uri askomics:faldoBegin ?gene1_start .
    ?gene1_uri askomics:faldoEnd ?gene1_end .
    ?Reference139_uri rdf:type <http://askomics.org/data/Reference> .
    ?Differential_Expression185_uri rdf:type <http://askomics.org/data/Differential
%20Expression> .
    ?Differential_Expression185_uri rdfs:label ?Differential_Expression1_Label .
    ?Contrast201_uri rdf:type <http://askomics.org/data/Contrast> .
    ?Contrast201_uri rdfs:label ?Contrast1_Label .
    ?Switch_A_B408_uri rdf:type <http://askomics.org/data/Switch%20A/B> .
    ?Switch_A_B408_uri askomics:faldoEnd ?Switch_A_B408_binend .
    ?Switch_A_B408_uri askomics:faldoBegin ?Switch_A_B408_binstart .
    ?Contrast542_uri rdf:type <http://askomics.org/data/Contrast> .
    ?Contrast542_uri rdfs:label ?Contrast201_Label .
    ?Atac_peak847_uri rdf:type <http://askomics.org/data/Atac%20peak> .
```

```
    ?Atac_peak847_uri askomics:faldoEnd ?Atac_peak847_PeakEnd .
    ?Atac_peak847_uri askomics:faldoBegin ?Atac_peak847_PeakStart .
    ?Condition948_uri rdf:type <http://askomics.org/data/Condition> .
    ?Condition948_uri <http://askomics.org/data/Days> ?Condition948_DaysCategory .
    FILTER ((?Switch_A_B408_binstart >= ?gene1_start && ?Switch_A_B408_binstart <= ?
gene1_end) || (?Switch_A_B408_binend >= ?gene1_start && ?Switch_A_B408_binend <= ?
gene1_end) || (?gene1_start >= ?Switch_A_B408_binstart && ?gene1_end <= ?
Switch_A_B408_binend))
    FILTER ((?Atac_peak847_PeakStart >= ?gene1_start && ?Atac_peak847_PeakStart <= ?
gene1_end) || (?Atac_peak847_PeakEnd >= ?gene1_start && ?Atac_peak847_PeakEnd <= ?
gene1_end) || (?gene1_start >= ?Atac_peak847_PeakStart && ?gene1_end <= ?
Atac_peak847_PeakEnd))
    VALUES ?Contrast1_Label { '2Qvs2W' } .
    VALUES ?Condition948_DaysCategory { <http://askomics.org/data/2> }
}
```

32- Number of genes that are implied in cast differentiation, are differentially expressed in Contrast 4Qvs4W, contain unique ATAC peak(s) and overlap with a Switch region

```
SELECT DISTINCT ?gene1_Label ?Differential_Expression1_Label ?Contrast1_Label ?
Contrast201_Label
WHERE {
    ?Reference139_uri <http://askomics.org/data/linked_to> ?gene1_uri .
    ?Differential_Expression185_uri <http://askomics.org/data/measured_in> ?gene1_uri .
    ?Differential_Expression185_uri <http://askomics.org/data/measured_in> ?Contrast201_uri .
    ?gene1_uri askomics:includeInReference ?block_1_408 .
    ?Switch_A_B408_uri askomics:includeInReference ?block_1_408 .
    ?Switch_A_B408_uri <http://askomics.org/data/measured_in> ?Contrast542_uri .
    ?gene1_uri askomics:includeInReference ?block_1_847 .
    ?Atac_peak847_uri askomics:includeInReference ?block_1_847 .
    ?Atac_peak847_uri <http://askomics.org/data/measured_in> ?Condition948_uri .
    ?gene1_uri rdf:type <http://askomics.org/data/gene> .
    ?gene1_uri rdfs:label ?gene1_Label .
    ?gene1_uri askomics:faldoBegin ?gene1_start .
    ?gene1_uri askomics:faldoEnd ?gene1_end .
    ?Reference139_uri rdf:type <http://askomics.org/data/Reference> .
    ?Differential_Expression185_uri rdf:type <http://askomics.org/data/Differential
%20Expression> .
    ?Differential_Expression185_uri rdfs:label ?Differential_Expression1_Label .
    ?Contrast201_uri rdf:type <http://askomics.org/data/Contrast> .
    ?Contrast201_uri rdfs:label ?Contrast1_Label .
    ?Switch_A_B408_uri rdf:type <http://askomics.org/data/Switch%20A/B> .
    ?Switch_A_B408_uri askomics:faldoEnd ?Switch_A_B408_binend .
    ?Switch_A_B408_uri askomics:faldoBegin ?Switch_A_B408_binstart .
    ?Contrast542_uri rdf:type <http://askomics.org/data/Contrast> .
    ?Contrast542_uri rdfs:label ?Contrast201_Label .
    ?Atac_peak847_uri rdf:type <http://askomics.org/data/Atac%20peak> .
    ?Atac_peak847_uri askomics:faldoEnd ?Atac_peak847_PeakEnd .
    ?Atac_peak847_uri askomics:faldoBegin ?Atac_peak847_PeakStart .
    ?Condition948_uri rdf:type <http://askomics.org/data/Condition> .
    ?Condition948_uri <http://askomics.org/data/Days> ?Condition948_DaysCategory .
```

```
    FILTER ((?Switch_A_B408_binstart >= ?gene1_start && ?Switch_A_B408_binstart <= ?
gene1_end) || (?Switch_A_B408_binend >= ?gene1_start && ?Switch_A_B408_binend <= ?
gene1_end) || (?gene1_start >= ?Switch_A_B408_binstart && ?gene1_end <= ?
Switch_A_B408_binend))
    FILTER ((?Atac_peak847_PeakStart >= ?gene1_start && ?Atac_peak847_PeakStart <= ?
gene1_end) || (?Atac_peak847_PeakEnd >= ?gene1_start && ?Atac_peak847_PeakEnd <= ?
gene1_end) || (?gene1_start >= ?Atac_peak847_PeakStart && ?gene1_end <= ?
Atac_peak847_PeakEnd))
    VALUES ?Contrast1_Label { '4Qvs4W' } .
    VALUES ?Condition948_DaysCategory { <http://askomics.org/data/4> }
}
```

33- Number of genes that are implied in cast differentiation, are differentially expressed in Contrast 2Qvs2W, contain unique Chip and ATAC peak(s)

```
SELECT DISTINCT ?gene1_Label ?Contrast1_Label
WHERE {
    ?Reference139_uri <http://askomics.org/data/linked_to> ?gene1_uri .
    ?gene1_uri askomics:includeInReference ?block_1_240 .
    ?Atac_peak240_uri askomics:includeInReference ?block_1_240 .
    ?Atac_peak240_uri <http://askomics.org/data/measured_in> ?Condition416_uri .
    ?gene1_uri askomics:includeInReference ?block_1_518 .
    ?Chip_peak518_uri askomics:includeInReference ?block_1_518 .
    ?Chip_peak518_uri <http://askomics.org/data/measured_in> ?Condition652_uri .
    ?Differential_Expression701_uri <http://askomics.org/data/measured_in> ?gene1_uri .
    ?Differential_Expression701_uri <http://askomics.org/data/measured_in> ?Contrast717_uri .
    ?gene1_uri rdf:type <http://askomics.org/data/gene> .
    ?gene1_uri rdfs:label ?gene1_Label .
    ?gene1_uri askomics:faldoBegin ?gene1_start .
    ?gene1_uri askomics:faldoEnd ?gene1_end .
    ?Reference139_uri rdf:type <http://askomics.org/data/Reference> .
    ?Atac_peak240_uri rdf:type <http://askomics.org/data/Atac%20peak> .
    ?Atac_peak240_uri askomics:faldoEnd ?Atac_peak240_PeakEnd .
    ?Atac_peak240_uri askomics:faldoBegin ?Atac_peak240_PeakStart .
    ?Condition416_uri rdf:type <http://askomics.org/data/Condition> .
    ?Condition416_uri <http://askomics.org/data/Days> ?Condition416_DaysCategory .
    ?Chip_peak518_uri rdf:type <http://askomics.org/data/Chip%20peak> .
    ?Chip_peak518_uri askomics:faldoBegin ?Chip_peak518_PeakStart .
    ?Chip_peak518_uri askomics:faldoEnd ?Chip_peak518_PeakEnd .
    ?Condition652_uri rdf:type <http://askomics.org/data/Condition> .
    ?Condition652_uri <http://askomics.org/data/Days> ?Condition416_DaysCategory .
    ?Differential_Expression701_uri rdf:type <http://askomics.org/data/Differential
%20Expression> .
    ?Contrast717_uri rdf:type <http://askomics.org/data/Contrast> .
    ?Contrast717_uri rdfs:label ?Contrast1_Label .
    FILTER ((?Atac_peak240_PeakStart >= ?gene1_start && ?Atac_peak240_PeakStart <= ?
gene1_end) || (?Atac_peak240_PeakEnd >= ?gene1_start && ?Atac_peak240_PeakEnd <= ?
gene1_end) || (?gene1_start >= ?Atac_peak240_PeakStart && ?gene1_end <= ?
Atac_peak240_PeakEnd))
    FILTER ((?Chip_peak518_PeakStart >= ?gene1_start && ?Chip_peak518_PeakStart <= ?
gene1_end) || (?Chip_peak518_PeakEnd >= ?gene1_start && ?Chip_peak518_PeakEnd <= ?
```

```
gene1_end) || (?gene1_start >= ?Chip_peak518_PeakStart && ?gene1_end <= ?
Chip_peak518_PeakEnd))
   VALUES ?Condition416_DaysCategory { <http://askomics.org/data/2> }
   VALUES ?Contrast1_Label { '2Qvs2W' } .
}
```

34- Number of genes that are implied in cast differentiation, are differentially expressed in Contrast 4Qvs4W, contain unique Chip and ATAC peak(s)

```
SELECT DISTINCT ?gene1_Label ?Contrast1_Label
WHERE {
   ?Reference139_uri <http://askomics.org/data/linked_to> ?gene1_uri .
   ?gene1_uri askomics:includeInReference ?block_1_240 .
   ?Atac_peak240_uri askomics:includeInReference ?block_1_240 .
   ?Atac_peak240_uri <http://askomics.org/data/measured_in> ?Condition416_uri .
   ?gene1_uri askomics:includeInReference ?block_1_518 .
   ?Chip_peak518_uri askomics:includeInReference ?block_1_518 .
   ?Chip_peak518_uri <http://askomics.org/data/measured_in> ?Condition652_uri .
   ?Differential_Expression1070_uri <http://askomics.org/data/measured_in> ?gene1_uri .
   ?Differential_Expression1070_uri <http://askomics.org/data/measured_in> ?Contrast1086_uri .
   ?gene1_uri rdf:type <http://askomics.org/data/gene> .
   ?gene1_uri rdfs:label ?gene1_Label .
   ?gene1_uri askomics:faldoBegin ?gene1_start .
   ?gene1_uri askomics:faldoEnd ?gene1_end .
   ?Reference139_uri rdf:type <http://askomics.org/data/Reference> .
   ?Atac_peak240_uri rdf:type <http://askomics.org/data/Atac%20peak> .
   ?Atac_peak240_uri askomics:faldoEnd ?Atac_peak240_PeakEnd .
   ?Atac_peak240_uri askomics:faldoBegin ?Atac_peak240_PeakStart .
   ?Condition416_uri rdf:type <http://askomics.org/data/Condition> .
   ?Condition416_uri <http://askomics.org/data/Days> ?Condition416_DaysCategory .
   ?Chip_peak518_uri rdf:type <http://askomics.org/data/Chip%20peak> .
   ?Chip_peak518_uri askomics:faldoBegin ?Chip_peak518_PeakStart .
   ?Chip_peak518_uri askomics:faldoEnd ?Chip_peak518_PeakEnd .
   ?Condition652_uri rdf:type <http://askomics.org/data/Condition> .
   ?Condition652_uri <http://askomics.org/data/Days> ?Condition416_DaysCategory .
   ?Differential_Expression1070_uri rdf:type <http://askomics.org/data/Differential
%20Expression> .
   ?Contrast1086_uri rdf:type <http://askomics.org/data/Contrast> .
   ?Contrast1086_uri rdfs:label ?Contrast1_Label .
   FILTER ((?Atac_peak240_PeakStart >= ?gene1_start && ?Atac_peak240_PeakStart <= ?
gene1_end) || (?Atac_peak240_PeakEnd >= ?gene1_start && ?Atac_peak240_PeakEnd <= ?
gene1_end) || (?gene1_start >= ?Atac_peak240_PeakStart && ?gene1_end <= ?
Atac_peak240_PeakEnd))
   FILTER ((?Chip_peak518_PeakStart >= ?gene1_start && ?Chip_peak518_PeakStart <= ?
gene1_end) || (?Chip_peak518_PeakEnd >= ?gene1_start && ?Chip_peak518_PeakEnd <= ?
gene1_end) || (?gene1_start >= ?Chip_peak518_PeakStart && ?gene1_end <= ?
Chip_peak518_PeakEnd))
   VALUES ?Condition416_DaysCategory { <http://askomics.org/data/4> }
   VALUES ?Contrast1_Label { '4Qvs4W' } .
}
```

35- Number of genes that are implied in cast differentiation, are differentially expressed in Contrast 2Qvs2W, contain unique Chip and ATAC peak(s) and overlap with Switch region

```
SELECT DISTINCT ?gene1_Label ?Contrast1_Label
WHERE {
    ?Reference139_uri <http://askomics.org/data/linked_to> ?gene1_uri .
    ?gene1_uri askomics:includeInReference ?block_1_240 .
    ?Atac_peak240_uri askomics:includeInReference ?block_1_240 .
    ?Atac_peak240_uri <http://askomics.org/data/measured_in> ?Condition416_uri .
    ?gene1_uri askomics:includeInReference ?block_1_518 .
    ?Chip_peak518_uri askomics:includeInReference ?block_1_518 .
    ?Chip_peak518_uri <http://askomics.org/data/measured_in> ?Condition652_uri .
    ?gene1_uri askomics:includeInReference ?block_1_821 .
    ?Switch_A_B821_uri askomics:includeInReference ?block_1_821 .
    ?Switch_A_B821_uri <http://askomics.org/data/measured_in> ?Contrast999_uri .
    ?gene1_uri rdf:type <http://askomics.org/data/gene> .
    ?gene1_uri rdfs:label ?gene1_Label .
    ?gene1_uri askomics:faldoBegin ?gene1_start .
    ?gene1_uri askomics:faldoEnd ?gene1_end .
    ?Reference139_uri rdf:type <http://askomics.org/data/Reference> .
    ?Atac_peak240_uri rdf:type <http://askomics.org/data/Atac%20peak> .
    ?Atac_peak240_uri askomics:faldoEnd ?Atac_peak240_PeakEnd .
    ?Atac_peak240_uri askomics:faldoBegin ?Atac_peak240_PeakStart .
    ?Condition416_uri rdf:type <http://askomics.org/data/Condition> .
    ?Condition416_uri <http://askomics.org/data/Days> ?Condition416_DaysCategory .
    ?Chip_peak518_uri rdf:type <http://askomics.org/data/Chip%20peak> .
    ?Chip_peak518_uri askomics:faldoBegin ?Chip_peak518_PeakStart .
    ?Chip_peak518_uri askomics:faldoEnd ?Chip_peak518_PeakEnd .
    ?Condition652_uri rdf:type <http://askomics.org/data/Condition> .
    ?Condition652_uri <http://askomics.org/data/Days> ?Condition416_DaysCategory .
    ?Switch_A_B821_uri rdf:type <http://askomics.org/data/Switch%20A/B> .
    ?Switch_A_B821_uri askomics:faldoEnd ?Switch_A_B821_binend .
    ?Switch_A_B821_uri askomics:faldoBegin ?Switch_A_B821_binstart .
    ?Contrast999_uri rdf:type <http://askomics.org/data/Contrast> .
    ?Contrast999_uri rdfs:label ?Contrast1_Label .
    FILTER ((?Atac_peak240_PeakStart >= ?gene1_start && ?Atac_peak240_PeakStart <= ?
gene1_end) || (?Atac_peak240_PeakEnd >= ?gene1_start && ?Atac_peak240_PeakEnd <= ?
gene1_end) || (?gene1_start >= ?Atac_peak240_PeakStart && ?gene1_end <= ?
Atac_peak240_PeakEnd))
    FILTER ((?Chip_peak518_PeakStart >= ?gene1_start && ?Chip_peak518_PeakStart <= ?
gene1_end) || (?Chip_peak518_PeakEnd >= ?gene1_start && ?Chip_peak518_PeakEnd <= ?
gene1_end) || (?gene1_start >= ?Chip_peak518_PeakStart && ?gene1_end <= ?
Chip_peak518_PeakEnd))
    FILTER ((?Switch_A_B821_binstart >= ?gene1_start && ?Switch_A_B821_binstart <= ?
gene1_end) || (?Switch_A_B821_binend >= ?gene1_start && ?Switch_A_B821_binend <= ?
gene1_end) || (?gene1_start >= ?Switch_A_B821_binstart && ?gene1_end <= ?
Switch_A_B821_binend))
    VALUES ?Condition416_DaysCategory { <http://askomics.org/data/2> }
    VALUES ?Contrast1_Label { '2Qvs2W' } .
}
```

36- Number of genes that are implied in cast differentiation, are differentially expressed in Contrast 4Qvs4W, contain unique Chip and ATAC peak(s) and overlap with Switch region

```
SELECT DISTINCT ?gene1_Label ?Contrast1_Label
WHERE {
   ?Reference139_uri <http://askomics.org/data/linked_to> ?gene1_uri .
   ?gene1_uri askomics:includeInReference ?block_1_240 .
   ?Atac_peak240_uri askomics:includeInReference ?block_1_240 .
   ?Atac_peak240_uri <http://askomics.org/data/measured_in> ?Condition416_uri .
   ?gene1_uri askomics:includeInReference ?block_1_518 .
   ?Chip_peak518_uri askomics:includeInReference ?block_1_518 .
   ?Chip_peak518_uri <http://askomics.org/data/measured_in> ?Condition652_uri .
   ?gene1_uri askomics:includeInReference ?block_1_821 .
   ?Switch_A_B821_uri askomics:includeInReference ?block_1_821 .
   ?Switch_A_B821_uri <http://askomics.org/data/measured_in> ?Contrast999_uri .
   ?gene1_uri rdf:type <http://askomics.org/data/gene> .
   ?gene1_uri rdfs:label ?gene1_Label .
   ?gene1_uri askomics:faldoBegin ?gene1_start .
   ?gene1_uri askomics:faldoEnd ?gene1_end .
   ?Reference139_uri rdf:type <http://askomics.org/data/Reference> .
   ?Atac_peak240_uri rdf:type <http://askomics.org/data/Atac%20peak> .
   ?Atac_peak240_uri askomics:faldoEnd ?Atac_peak240_PeakEnd .
   ?Atac_peak240_uri askomics:faldoBegin ?Atac_peak240_PeakStart .
   ?Condition416_uri rdf:type <http://askomics.org/data/Condition> .
   ?Condition416_uri <http://askomics.org/data/Days> ?Condition416_DaysCategory .
   ?Chip_peak518_uri rdf:type <http://askomics.org/data/Chip%20peak> .
   ?Chip_peak518_uri askomics:faldoBegin ?Chip_peak518_PeakStart .
   ?Chip_peak518_uri askomics:faldoEnd ?Chip_peak518_PeakEnd .
   ?Condition652_uri rdf:type <http://askomics.org/data/Condition> .
   ?Condition652_uri <http://askomics.org/data/Days> ?Condition416_DaysCategory .
   ?Switch_A_B821_uri rdf:type <http://askomics.org/data/Switch%20A/B> .
   ?Switch_A_B821_uri askomics:faldoEnd ?Switch_A_B821_binend .
   ?Switch_A_B821_uri askomics:faldoBegin ?Switch_A_B821_binstart .
   ?Contrast999_uri rdf:type <http://askomics.org/data/Contrast> .
   ?Contrast999_uri rdfs:label ?Contrast1_Label .
   FILTER ((?Atac_peak240_PeakStart >= ?gene1_start && ?Atac_peak240_PeakStart <= ?
gene1_end) || (?Atac_peak240_PeakEnd >= ?gene1_start && ?Atac_peak240_PeakEnd <= ?
gene1_end) || (?gene1_start >= ?Atac_peak240_PeakStart && ?gene1_end <= ?
Atac_peak240_PeakEnd))
   FILTER ((?Chip_peak518_PeakStart >= ?gene1_start && ?Chip_peak518_PeakStart <= ?
gene1_end) || (?Chip_peak518_PeakEnd >= ?gene1_start && ?Chip_peak518_PeakEnd <= ?
gene1_end) || (?gene1_start >= ?Chip_peak518_PeakStart && ?gene1_end <= ?
Chip_peak518_PeakEnd))
   FILTER ((?Switch_A_B821_binstart >= ?gene1_start && ?Switch_A_B821_binstart <= ?
gene1_end) || (?Switch_A_B821_binend >= ?gene1_start && ?Switch_A_B821_binend <= ?
gene1_end) || (?gene1_start >= ?Switch_A_B821_binstart && ?gene1_end <= ?
Switch_A_B821_binend))
   VALUES ?Condition416_DaysCategory { <http://askomics.org/data/4> }
   VALUES ?Contrast1_Label { '4Qvs4W' } .
}
```
