# Supplementary material for: Generic and queryable data integration schema for transcriptomics and epigenomics studies
Source: Comput Struct Biotechnol J. 2024 Nov 19;23:4232–41. doi: 10.1016/j.csbj.2024.11.022 (PMC11629147; doi:10.1016/j.csbj.2024.11.022)
Supplement: MMC 3 — SPARQL query to retrieve SemOpenAlex identifiers. [file mmc3.pdf]

SemOpenAlex SPARQL

```
PREFIX rdf: <http://www.w3.org/1999/02/22-rdf-syntax-ns#>
PREFIX rdfs:<http://www.w3.org/2000/01/rdf-schema#>
PREFIX owl: <http://www.w3.org/2002/07/owl#>
PREFIX xsd: <http://www.w3.org/2001/XMLSchema#>
PREFIX dc: <http://purl.org/dc/elements/1.1/>
PREFIX dcterms: <http://purl.org/dc/terms/>
PREFIX skos: <http://www.w3.org/2004/02/skos/core#>
PREFIX foaf: <http://xmlns.com/foaf/0.1/>

PREFIX sh: <http://www.w3.org/ns/shacl#>

PREFIX cito: <http://purl.org/spar/cito/>
PREFIX fabio: <http://purl.org/spar/fabio/>
#PREFIX prism: <http://prismstandard.org/namespaces/basic/2.0/>
PREFIX prism: <http://purl.org/spar/datacite/>
PREFIX pubmed: <https://pubmed.ncbi.nlm.nih.gov/>

PREFIX soa: <https://semopenalex.org/ontology/>
#PREFIX soa: <https://semopenalex.org/property/>
PREFIX soaclass: <https://semopenalex.org/class/>
PREFIX soaproperty: <https://semopenalex.org/property/>
PREFIX soaconcept: <https://semopenalex.org/concept/>
PREFIX soawork: <https://semopenalex.org/work/>

SELECT * WHERE {
  VALUES ?DOI {
        "https://doi.org/10.1073/pnas.96.10.5575"
    "https://doi.org/10.3389/fgene.2018.00416"
        "https://doi.org/10.1098/rspb.2021.2663"
        "https://doi.org/10.1371/journal.pone.0013455"
        "https://doi.org/10.1242/jeb.061499"
        "https://doi.org/10.1186/1471-213x-7-70"
        "https://doi.org/10.1371/journal.pone.0013455"
        "https://doi.org/10.1038/srep10694"
        "https://doi.org/10.1007/s00441-001-0490-y"
        "https://doi.org/10.1093/jis/5.1.36"
        "https://doi.org/10.1007/s10528-008-9202-6"
        "https://doi.org/10.1021/pr200473a"
        "https://doi.org/10.3390/insects12070649"
        "https://doi.org/10.1016/j.jinsphys.2016.03.004"
        "https://doi.org/10.1038/nature10093"
        "https://doi.org/10.1016/j.celrep.2020.108580"
        "https://doi.org/10.1038/nature10093"
        "https://doi.org/10.1371/journal.pone.0022195"
        "https://doi.org/10.1021/pr200473a"
        "https://doi.org/10.1021/pr200473a"
        "https://doi.org/10.1371/journal.pone.0013455"
        "https://doi.org/10.1021/pr200473a"
        "https://doi.org/10.1038/nature10093"
```

```
        "https://doi.org/10.1038/nature10093"
        "https://doi.org/10.1021/pr200473a"
        "https://doi.org/10.1038/srep10694"
        "https://doi.org/10.1111/imb.12114"
        "https://doi.org/10.1021/pr200473a"
        "https://doi.org/10.3389/fgene.2018.00416"
        "https://doi.org/10.1016/j.jprot.2012.10.012"
        "https://doi.org/10.1016/j.cub.2019.05.059"
        "https://doi.org/10.1371/journal.pone.0013455"
        "https://doi.org/10.3389/fgene.2018.00416"
        "https://doi.org/10.1021/pr200473a"
        "https://doi.org/10.1021/pr800823r"
        "https://doi.org/10.3389/fcell.2020.00361"
        "https://doi.org/10.1371/journal.pgen.1009801"
        "https://doi.org/10.1101/gr.236497.118"
        "https://doi.org/10.3389/fgene.2018.00416"
        "https://doi.org/10.1002/ece3.7125"
        "https://doi.org/10.1186/1471-2164-14-903"
        "https://doi.org/10.1111/mec.15047"
        "https://doi.org/10.7554/elife.45009"
        "https://doi.org/10.3390/ijms222212105"
        "https://doi.org/10.1590/1678-4685-gmb-2020-0173"
        "https://doi.org/10.1371/journal.pone.0013455"
        "https://doi.org/10.1016/j.exger.2015.08.001"
        "https://doi.org/10.1016/j.jinsphys.2008.04.021"
        "https://doi.org/10.1016/0022-1910(90)90121-U"
        "https://doi.org/10.1371/journal.pone.0013455"
        "https://doi.org/10.3389/fgene.2018.00416"
        "https://doi.org/10.1371/journal.pone.0040111"
        "https://doi.org/10.1016/j.cub.2019.05.059"
        "https://doi.org/10.1016/j.cub.2019.05.059"
        "https://doi.org/10.1021/pr800823r"
        "https://doi.org/10.1021/pr800823r"
        "https://doi.org/10.1038/nature05260"
        "https://doi.org/10.1371/journal.pone.0102663"
        "https://doi.org/10.1101/gad.343699.120"
        "https://doi.org/10.1186/gb-2003-4-10-r62"
        "https://doi.org/10.1371/journal.pone.0013455"
        "https://doi.org/10.1038/srep10694"
        "https://doi.org/10.1111/1744-7917.12788"
        "https://doi.org/10.3389/fgene.2018.00416"
        "https://doi.org/10.1038/nature05260"

  }
  ?Article prism:doi ?DOI .

}
```
